# Mono- and Dimeric Naphthalenones from the Marine-Derived Fungus *Leptosphaerulina chartarum* 3608

**DOI:** 10.3390/md16050173

**Published:** 2018-05-21

**Authors:** Panpan Zhang, Chunxiu Jia, Jiajia Lang, Jing Li, Guangyuan Luo, Senhua Chen, Sujun Yan, Lan Liu

**Affiliations:** 1 School of Marine Sciences, Sun Yat-sen University, Guangzhou 510006, China; zhangpp23@mail2.sysu.edu.cn (P.Z.); Lijing356@mail.sysu.edu.cn (J.L.); luogy331@gmail.com (G.L.); cesllan@mail.sysu.edu.cn (L.L.); 2 School of Chemistry, Sun Yat-sen University, Guangzhou 510006, China; jiachx@mail2.sysu.edu.cn (C.J.); langjj@mail2.sysu.edu.cn (J.L.); 3 Key Laboratory of Functional Molecules from Oceanic Microorganisms (Sun Yat-sen University), Department of Education of Guangdong Province, Guangzhou 510080, China

**Keywords:** *Leptosphaerulina chartarum*, naphthalenones, isocoumarine, anti-inflammatory

## Abstract

Five new naphthalenones, two enantiomers (−)-**1** and (+)-**1** leptothalenone A, (−)-4,8-dihydroxy-7-(2-hydroxy-ethyl)-6-methoxy-3,4-dihydro-2*H*-naphthalen-1-one ((−)-**2**), (4*S*, 10*R*, 4’*S*)-leptotha-lenone B (**5**), (4*R*, 10*S*, 4’*S*)-leptothalenone B (**6**), and a new isocoumarine, 6-hydroxy-5,8-dimethoxy-3-methyl-1*H*-isochromen-1-one (**4**), along with two known compounds (+)-4,8-dihydroxy-7-(2-hydroxy-ethyl)-6-methoxy-3,4-dihydro-2*H*-naphthalen-1-one ((+)-**2**) and (+)-10-norparvulenone (**3**) were isolated from the marine-derived fungus *Leptosphaerulina chartarum* 3608. The structures of new compounds were elucidated by HR-ESIMS, NMR, and ECD analysis. All compounds were evaluated for cytotoxicity and anti-inflammatory activity. Compound **6** showed moderate anti-inflammatory activity by inhibiting the production of nitric oxide (NO) in lipopolysaccharide-stimulated RAW264.7 cells, with an IC_50_ value of 44.5 μM.

## 1. Introduction

Marine-derived micro-organisms are known as a rich source of natural products with novel structure and interesting bioactivities [1]. A large number of the new compounds from marine-derived fungi are discovered every year. Among these, naphthalenone derivatives are side products of the 1,8-dihydroxynaphthalene polyketide pathway [2], which are common in the secondary metabolites from fungi with diverse bioactivities, such as antimicrobial [3], antiviral [4], cytotoxic [5], and antimalarial activities [3]. Besides, naphthalenone derivatives have structural diversity not only in the planar structure but also in the absolute configuration. Abdel-Latef et al. reported a pair epimer of (3R*,4S*)-3,4-dihydroxy-7-methyl-3,4-dihydro-1(2*H*)-naphthalenone and (3S*,4S*)-3,4-dihydroxy-7-methyl-3,4-dihydro-1(2*H*)-naphthalenone from the Algicolous marine fungus *Acremonium* sp. [6]. Wang et al. isolated a pair of naphthalenone enantiomers, corynenones A and B, from sponge-derived fungus *Corynespora cassiicola* [7].

**Figure 1:**
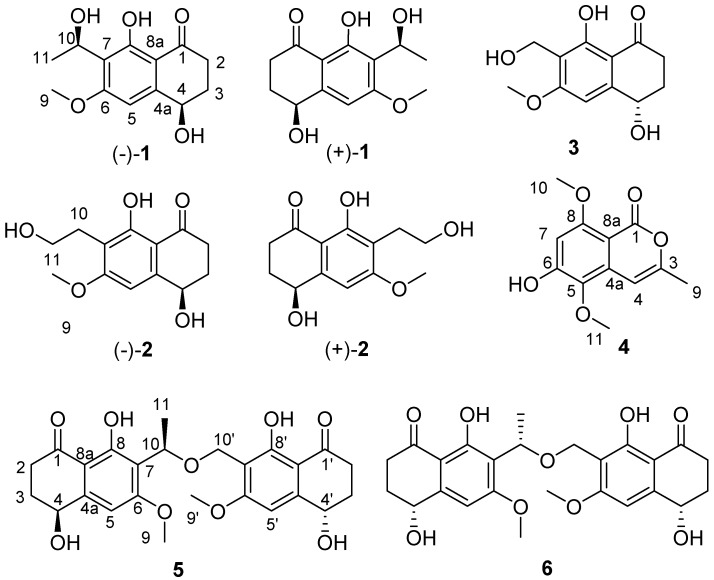
Chemical structures of **1**–**6**.

During our ongoing research on secondary metabolites from marine-derived fungus, five new naphthalenones, two enantiomers (−)-**1** and (+)-**1** leptothalenone A, (−)-4,8-dihydroxy-7-(2-hydroxy-ethyl)-6-methoxy-3,4-dihydro-2*H*-naphthalen-1-one ((−)-**2**), (4*S*, 10*R*, 4′*S*)-leptothalenone B (**5**), (4*R*, 10*S*, 4′*S*)-leptothalenone B (**6**), and a new isocoumarine (**4**), along with two known compounds (+)-4,8-dihydroxy-7-(2-hydroxy-ethyl)-6-methoxy-3,4-dihydro-2*H*-naphthalen-1-one ((+)-**2**) and (+)-10-norparvulenone (**3**) (Figure 1) were isolated from *Leptosphaerulina chartarum* 3608 from a crinoid collected in Xuwen, Zhanjiang City, Guangdong Province, China. In this paper, we described the structure determination of the new compounds as well as the bioactivity assay of these isolated compounds from the CHCl_3_ extract of *L. chartarum* 3608.

## 2. Results and Discussion

Compound (±)-**1** was obtained as a light brown powder and had a molecular formula C_13_H_16_O_5_ according to the positive HR-ESIMS ion at *m*/*z* 275.0885 [M + Na]^+^ (calculated for C_13_H_16_O_5_Na, 275.0890), indicating six degrees of unsaturation. The ^1^H NMR data (Table 1) revealed the presence of one five-substituted aromatic proton [*δ*_H_ 6.79 (1H, s, H-5)], one methoxyl [*δ*_H_ 3.92 (3H, s, H-9)], one methyl [*δ*_H_ 1.48 (3H, d, *J* = 6.8, H-11)], two methenes [*δ*_H_ 2.62 (1H, m, H-2), *δ*_H_ 2.79 (1H, m, H-2), *δ*_H_ 2.01 (1H, m, H-3), *δ*_H_ 2.26 (1H, m, H-2)], two oxygenated methines [*δ*_H_ 4.77 (1H, dd, *J* = 8.9, 3.9, H-4), *δ*_H_ 5.31 (1H, s, H-11)]. The ^13^C NMR and DEPT spectra displayed a ketone carbonyl (*δ*_C_ 204.6), six aromatic carbons (*δ*_C_ 164.8, 162.7, 150.2, 119.1, 111.1*,* 102.2), an oxygenated methyl (*δ*_C_ 56.4), two oxygenated methine (*δ*_C_ 68.6 and 63.4), and two methylenes (*δ*_C_ 36.1 and 32.7). Further analyses of the NMR data showed that compound **1** shared a good similarity with the known compound *O*-methylasparvenone [8], except for an extra hydroxyl group which was connected to C-10 based on the chemical shift values (*δ*_H/C_ 5.31 and 63.4, H-10 and C-10) and the ^1^H-^1^H COSY correlation of H-10 and H-11, as well as the HMBC correlation of H-11 with C-10 and C-7. Thus, the planar structure of **1** was successfully assigned as 9-hydroxyl-*O*-methylasparvenone (Figure 1). 

**Figure 2:**
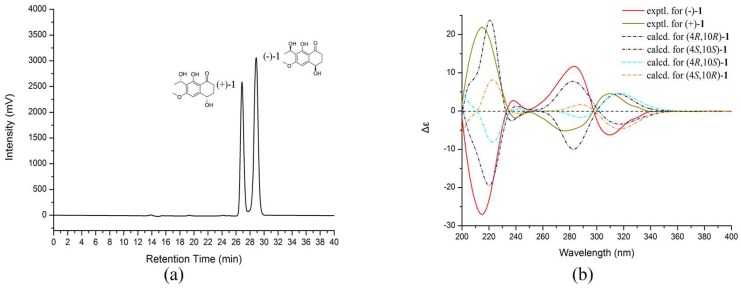
Chiral HPLC chromatogram (**a**); experimental and calculation ECD spectra (**b**) of (±)-**1**.

Compound **1** was of racemic nature because it lacked any CD (circular dichroism) maximum and optical rotation. Subsequent chiral HPLC purification of (±)-**1** led to the separation of the two enantiomers, (−)-**1** and (+)-**1** (Figure 2a), which showed opposite optical rotations [(−)-**1** (${\left[\alpha \right]}_{D}^{20}$ = −24.8, *c* 0.47, MeOH) and (+)-**1** (${\left[\alpha \right]}_{D}^{20}$ = 27.9, *c* 0.30, MeOH)] and opposite Cotton effects in their CD spectra (Figure 2b). The experimental ECD spectra of (−)-**1** exhibited negative Cotton effects (CEs) at 214, 310 nm, and positive CEs at 238, 282 nm, while (+)-**1** exhibited positive CEs at 214, 310 nm, and negative CEs at 238, 282 nm. To determine the absolute configuration of (−)-**1** and (+)-**1**, their theoretical ECD spectra of four possible configurations [(4*R*,10*R*)-**1**, (4*R*,10*S*)-**1**, (4*S*,10*R*)-**1**, (4*S*,10*S*)-**1**] were calculated by a quantum chemical method at the [B3LYP/6-311+G(2d,p)] level and are shown in Figure 2b. The predicted ECD curve of (4*R*,10*R*)-**1** was in accordance with the experimental ECD curve of (−)-**1**, and (4*S*,10*S*)-**1** was also in agreement with the experimental ECD curve of (+)-**1** (Figure 2b). This result suggested that the absolute configuration of (−)-**1** was 4*R*, 10*R* and (+)-**1** was 4*S*, 10*S*. Therefore, (±)-**1** was named as (±)-leptothalenone A.

**Figure 3:**
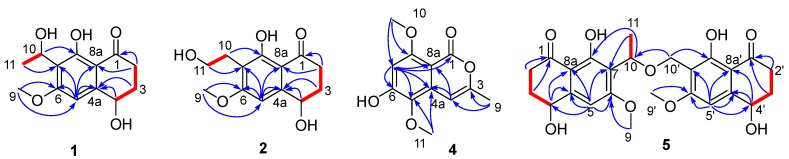
Key ^1^H-^1^H COSY (red line) and HMBC (blue arrow) correlations of compounds **1**, **2**, **4** and **5**.

**Figure 4:**
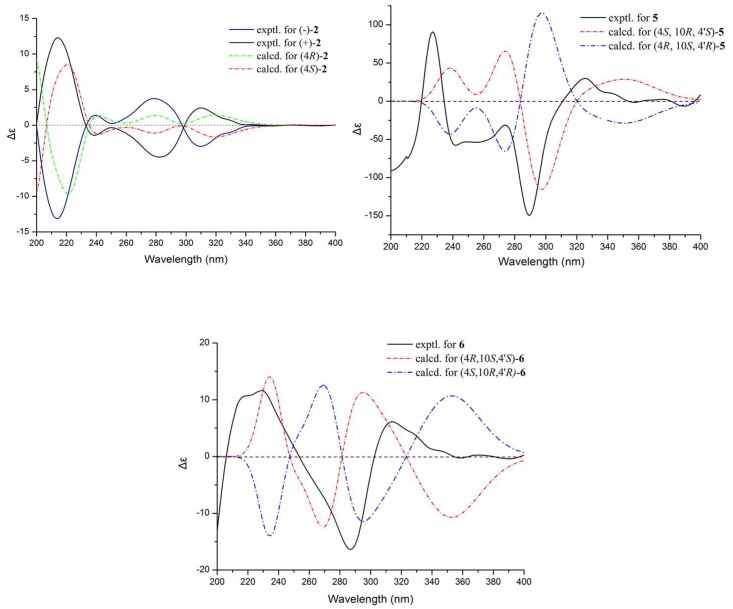
Experimental and calculation ECD spectra of **2**, **5** and **6**.

Compound (±)-**2** was obtained as a light yellow powder. Its molecular formula was determined as C_13_H_16_O_5_ according to the positive HR-ESIMS ([M + Na]^+^ *m*/*z* 275.0885, calculated for C_13_H_16_O_5_Na) and was the same as (±)-leptothalenone A. Through analysis of the NMR data of (±)-**2** (Table 1 and Table 2, Figure 3), the planar structure of (±)-**2** was identified as the known compound 4,8-dihydroxy-7-(2-hydroxy-ethyl)-6-methoxy-3,4-dihydro-2*H-*naphthalen-1-one with positive optical rotation (${\left[\alpha \right]}_{D}^{20}$ = +28.0, *c* 0.1, MeOH) [9], whose configuration has never been determined. Using the similar chiral HPLC purification method, (±)-**2** was separated into two enantiomers, (−)-**2** and (+)-**2**, which displayed opposite optical rotations [(−)-**2** (${\left[\alpha \right]}_{D}^{20}$ = −14.0, *c* 0.10, MeOH) and (+)-**2** (${\left[\alpha \right]}_{D}^{20}$ = 15.1, *c* 0.07, MeOH)] and opposite Cotton effects in their CD spectra. The calculated ECD spectra of (4*R*)-**2** and (4*S*)-**2** agreed well with the experimental ECD spectra of (−)-**2** and (+)-**2** (Figure 4). Thus, the configuration of known compound (+)-**2** was assigned as 4*R*, while the enantiomer (−)-**2** was a new compound with the configuration of 4*S*. 

Compound **4** was obtained as a white crystal. The HR-ESIMS result (*m*/*z* 237.0812 [M + H]^+^) suggested the molecular formula of **4** was C_12_H_12_O_5_ with seven degrees of unsaturation. The 1D and 2D NMR data indicated that compound **4** shared the same isocoumarin skeleton as 6,8-dihydroxy-5-methoxy-3-methyl-1*H*-isochromen-1-one [10]. The only difference between them was that the hydroxyl group (8-OH) of the known compound was replaced by a methoxyl group (8-OCH_3_) of **4**, which was further confirmed by the HMBC correlation of H-10 to C-5 and C-6. Hence, compound **4** was assigned as 6-hydroxy-5,8-dimethoxy-3-methyl-1*H*-isochromen-1-one.

Compounds **5** and **6** were both isolated as a light brown amorphous powder from the same fraction using the RP-HPLC [MeOH-H_2_O 60:40, t_R_ (**5**) = 52 min, t_R_ (**6**) = 56 min], implying they were epimers. The different optical rotation [${\left[\alpha \right]}_{D}^{20}$ = −11.9 (MeOH, *c* 0.21) of **5**, [${\left[\alpha \right]}_{D}^{20}$ = 11.4 (MeOH, *c* 0.21) of **6**] indicated that **6** was a stereoisomer of **5**. They had the same molecular formula C_25_H_28_O_9_, which was determined by HR-ESIMS at *m*/*z* 495.1628 (calculated for C_25_H_28_O_9_Na, 495.1626), corresponding to 12 degrees of unsaturation. The IR spectrum displayed absorption at 3383 cm^−1^ indicative of the existence of hydroxyl group. The ^1^H and ^13^C NMR spectra of compounds **5** and **6** revealed the presence of 24 protons and 25 carbons (two ketone carbonyls, ten aromatic quaternary carbons, two aromatic methines, three methyls, five methylenes, three methine). There were 14 sp^2^ carbon signals (two of which were carbonyls) that appeared pairwise in ^13^C NMR spectrum indicating that **5** and **6** were heterodimers of naphthalenones. Detailed analysis of the ^1^H-^1^H COSY and HMBC spectra (Figure 3), compounds **5** and **6** had the same planar structures as two monomeric units leptothalenone A (**1**) and 10-norparvulenone (**3**). The two monomeric units were connected through an ether bond (10-O-10’) based on the HMBC correlations from H-10′ to C-7′ and C-10. Therefore, the planar structures of **5** and **6** were defined as 1,5-dihydroxy-3-methoxy-8-oxo-5,6,7,8-tetrahydronaphthalen-2-yl)ethoxy)methyl)-4,8-dihydroxy-6-methoxy-3,4-dihydronaphthalen-1(2*H*)-one. 

The absolute configurations of **5** and **6** were established by comparing the experimental ECD data with the calculated values, in combination with biosynthetic considerations. Among the isolated monomeric naphthalenones, (+)-10-norparvulenone (**3**) was only obtained as an enantiomerically pure compound with *S* configuration, supporting by the X-ray single-crystal diffraction (Figure S45). The biosynthetic pathway suggests that the configurations at C-4’ of **5** and **6** were the same as those of the monomeric 10-norparvulenone (**3**). The ECD of the remaining four configurations [(4*R*, 10*S*, 4′*S*) (4*S*, 10*R*, 4′*S*) (4*R*, 10*R*, 4′*S*) (4*S*, 10*S*, 4′*S*)] was calculated by a quantum chemical method at the [B3LYP/6 − 311 + g(2d,p)] level. The theoretical ECD curve of (4*S*, 10*R*, 4′*S*) agreed well with the experimental ECD curve of (−)-**5**, and (4*R*, 10*S*, 4′*S*) was also in agreement with the experimental ECD curve of (+)-**6** (Figure 4).

The known compound, (+)-10-norparvulenone (**3**) was identified by NMR, MS and optical rotation data analysis and comparison of spectroscopic data with the literature [9]. The optical rotation of 10-norparvulenone was [${\left[\alpha \right]}_{D}^{20}$ = −27.0 (MeOH, *c* 0.21), and that of **3** was [${\left[\alpha \right]}_{D}^{20}$ = +16.6 (MeOH, *c* 0.21), which indicated their absolute configurations were opposite. The absolute configuration of **3** was 4*S* supported by X-ray single-crystal diffraction using anomalous scattering of Cu Kα radiation with Flack parameter = 0.00(6) (Figure S45). Thus, compound **3** was named as (+)-10-norparvulenone. 

All the compounds were tested for their inhibition activity against LPS-activated NO production in RAW264.7 cells using the Griess assay. Compound **6** displayed moderate inhibitory effects on the production of NO with an IC_50_ value of 44.5 ± 1.1 μM, compared to the positive control indomethacin (IC_50_ = 37.5 ± 1.6 μM), while the other compounds showed no significant anti-inflammatory activity (IC_50_ > 100 μM). In addition, some of naphthalenones have been found to have cytotoxic activity according to previous studies [2,5,11,12]. Thus, all the isolated compounds were evaluated for their cytotoxicity against A549 (lung cancer), HeLa (cervical cancer), and MCF-7 (breast cancer) human cancer cell lines using MTT assay and displayed no cytotoxicity against all three cell lines at 50 μM. 

## 3. Materials and Methods

### 3.1. General Experimental Procedures

Optical rotations were measured on an MCP 200 polarimeter by using an Na lamp (Shimadzu). UV spectra were obtained on a Blue Star A spectrophotometer. A Fourier transformation infra-red spectrometer coupled with infra-red microscope EQUINOX 55 (Bruker, Rheinstetten, Germany) was used to record the IR spectra. NMR spectra were obtained on a Bruker Avance 400 MHz spectrometer with tetramethylsilane as the internal standard. HR-ESIMS data were obtained on a LTQ-Orbitrap LC-MS spectrometer (Thermo Corporation, Waltham, MA, USA). ESIMS spectra were obtained on an ACQUITY QDA (Waters Corporation, Milford, MA, USA). HPLC was carried out on an Essentia LC-16 with an SPD-16 Detector (Shimadzu, Shanghai, China). Column chromatography was carried out on silica gel (100–200 mesh, 200–300 mesh, Qing dao Marine Chemical Factory, Qingdao, China) and Sephadex LH-20 (GE Healthcare, Littile Chalfont, UK). 

### 3.2. Fungal Material

The fungal strain 3608 was isolated from a crinoid collected in Xuwen, Zhanjiang City, Guangdong Province, China, in August 2014. It was identified as *L. chartarum* by ITS sequence, and the sequence data have been submitted to and deposited in the GenBank database under accession number MF980969. The fungal strain has been preserved at the school of marine science, Sun Yat-Sen University.

### 3.3. Extraction, Isolation, and Characterization

The fungus *L. chartarum* 3608 was cultured at room temperature (25–30 °C) for one month in 1000 mL Erlenmeyer flasks containing rice medium, composed of 60 mL rice, 80 mL H_2_O, and 3% sea salt. After 30 days of cultivation, the fermented rice substrate of 170 flasks was extracted three times with MeOH to yield the organic extract (70 g). The organic extract was portioned into three phases by successive extraction with n-hexane (43 g), CHCl_3_ (8 g), EtOAc (17 g). 

The CHCl_3_ phase was subjected to a silica gel column (6 × 12 cm, 100–200 mesh) and was eluted with PE-EtOAc (*v*/*v*, 80:20, 70:30, 60:40, 50:50, 40:60, 20:80 and 0:100, 400 × 5 mL each gradient) to yield seven fractions (A–F). Fr. D was separated by silica gel CC (3.5 × 12 cm, 200–300 mesh), eluted with PE-EtOAc (*v*/*v* 70:30, 60:40, 65:45, 50:50, 55:45 and 30:70) and combined with the same fractions based on the TLC results to yield Fr.D.1–Fr.D.5. Fr.D.1 was further subjected to silica gel CC (2 × 12 cm, 200–300 mesh) by isocratic elution by PE-EtOAc (65:35) to yield compound **4** (6 mg). After recrystallization, the racemates of **1** and **2** were obtained from Fr.D.2 and Fr.D.3, respectively. The racemate of **1** was separated by chiral PR-HPLC (30% CH_3_CN-H_2_O, flow rate 1 mL/min, Ultimate Amy-SR column 10 × 250 mm, 5 μm) to yield (−)-**1** (5 mg), (+)-**1** (5 mg). The racemate of **2** was also separated by chiral PR-HPLC (30% CH_3_CN-H_2_O, flow rate 1 mL/min, Ultimate Amy-SR column 10 × 250 mm, 5 μm) to yield (−)-**2** (7 mg), and (+)-**2** (7 mg). Fr.E was subjected to silica gel CC (3.5 × 12 cm, 200–300 mesh) and eluted with PE-EtOAc (40:60) to yield **3** (300 mg). Fr.F was subjected to RP-C18 (3.5 × 12 cm), eluted with 60% MeOH-H_2_O, and then purified by PR-HPLC (60% MeOH-H_2_O, flow rate 1 mL/min, Ultimate XB-C18 column 10 × 250 mm, 5 μm) to yield **5** (3 mg) and **6** (2 mg), whose retention times were 52 min and 56 min, respectively. 

#### 3.3.1. (−)-Leptothalenone A ((−)-**1**)

Light brown powder; [${\left[\alpha \right]}_{D}^{20}$ = −24.8 (MeOH, *c* 0.47); UV (MeOH) *λ*_max_ (log *ε*) 222 (4.00), 286 (3.92) nm; CD (MeOH) *λ*_max_ (*Δε*) 214 (−26.9), 238 (+2.74), 282 (+11.6), 310 (−6.24) nm; IR (neat) *ν*_max_ 3381, 2923, 1626, 1416, 1286, 1219, 1157, 1080, 843 cm^−1^; ^1^H NMR (400 MHz, MeOD) and ^13^C NMR (100 MHz, MeOD) data, see Table 1 and Table 2; HR-ESIMS *m*/*z* 275.0885 [M + Na]^+^ (calculated for C_13_H_16_O_5_Na, 275.0890).

#### 3.3.2. (+)-Leptothalenone A ((+)-**1**)

Light brown powder; [${\left[\alpha \right]}_{D}^{20}$ = 27.9 (MeOH, *c* 0.30); UV (MeOH) *λ*_max_ (log *ε*) 222 (3.91), 286 (3.79) nm; CD (MeOH) *λ*_max_ (*Δε*) 214 (+21.8), 238 (−1.75), 282 (−5.20), 310 (+4.54) nm; IR (neat) *ν*_max_ 3381, 2923, 1626, 1416, 1286, 1219, 1157, 1080, 843 cm^−1^; ^1^H NMR (400 MHz, MeOD) and ^13^C NMR (100 MHz, MeOD) data, see Table 1 and Table 2; HR-ESIMS *m*/*z* 275.0885 [M + Na]^+^ (calculated for C_13_H_16_O_5_Na, 275.0890).

#### 3.3.3. (−)-4,8-Dihydroxy-7-(2-hydroxy-ethyl)-6-methoxy-3,4-dihydro-2*H*-naphthalen-1-one ((−)-**2**)

White powder; [${\left[\alpha \right]}_{D}^{20}$ = −14.0 (MeOH, *c* 0.10); UV (MeOH) *λ*_max_ (log *ε*) 224 (4.26), 287 (3.17) nm; CD (MeOH) λ_max_ (*Δε*) 213 (−13.1), 239 (1.40), 279 (3.75) nm; IR (neat) ν_max_ 3374, 2945, 1622, 1417, 1292, 1211, 1138, 1092, 831 cm^−1^; ^1^H NMR (400 MHz, MeOD) and ^13^C NMR (100 MHz, MeOD), see Table 1 and Table 2; HR-ESIMS *m/z* 275.0885 [M + Na]^+^ (calculated for C_13_H_16_O_5_Na, 275.0890). 

#### 3.3.4. (+)-4,8-Dihydroxy-7-(2-hydroxy-ethyl)-6-methoxy-3,4-dihydro-2*H*-naphthalen-1-one ((+)-**2**)

White powder; [${\left[\alpha \right]}_{D}^{20}$ = 15.1 (MeOH, *c* 0.07); UV (MeOH) *λ*_max_ (log *ε*) 224 (4.26), 287 (3.17) nm; CD (MeOH) *λ*_max_ (*Δ*ε) 214 (+12.3), 230 (−1.42), 282 (−4.47); IR (neat) *ν*_max_ 3374, 2945, 1622, 1417, 1292, 1211, 1138, 1092, 831 cm^−1^; ^1^H NMR (400 MHz, MeOD) *δ* 2.59 (ddd, *J* = 17.7, 10.1, 4.6 Hz, H-2a); 2.77 (ddd, *J* = 17.7, 6.4, 4.6 Hz, H-2b); 2.01 (m, H-3a); 2.24 (m, H-3b); 4.76 (dd, *J* = 8.7, 3.9 Hz, H-4); 6.75 (s, H-5); 2.86 (m, H-10); 3.56 (t, *J* = 7.5 Hz, H-11); 3.90 (s, H-9); ^13^C NMR (100 MHz, MeOD) *δ* 203.0 (C, C-1); 34.5 (CH_2_, C-2); 31.4 (CH_2_, C-3); 67.2 (CH, C-4); 147.7 (CH_2_, C-4a); 100.4 (CH, C-5); 164.1 (C, C-6); 111.9 (C, C-7); 162.0 (C, C-8); 109.6 (C, C-8a); 25.3 (CH_2_, C-10); 60.1 (CH_2_, C-11); 55.0 (CH_3_, C-9); HR-ESIMS *m*/*z* 275.0885 [M + Na]^+^ (calculated for C_13_H_16_O_5_Na, 275.0890).

#### 3.3.5. (+)-10-Norparvulenone (**3**)

Yellow powder; [${\left[\alpha \right]}_{D}^{20}$ = 16.6 (MeOH *c* 0.14); UV (MeOH) *λ*_max_ (log *ε*) 224(4.32), 285 (4.18); 320 (3.72) nm; CD (MeOH) *λ*_max_ (*Δε*) 216 (+18.4), 240 (−3.14), 274 (−6.20), 305 (4.75) nm; IR (neat) *ν*_max_ 3379, 2939, 1616, 1416, 1290, 1132, 993, 829 cm^−1^; ^1^H NMR (400 MHz, MeOD) *δ* 2.61 (ddd, *J* = 12.9, 8.2, 4.6 Hz, H-2a); 2.76 (ddd, *J* = 15.6, 8.5, 4.6 Hz, H-2b); 2.01 (m, H-3a); 2.25 (m, H-3b); 4.77 (dd, *J* = 9.0, 3.8 Hz, H-4); 6.77 (s, H-5); 4.63 (s, H-9); 3.92 (s, 6-OCH_3_); ^13^C NMR (100 MHz, MeOD) *δ* 204.6 (C, C-1); 36.3 (CH_2_, C-2); 33.0 (CH_2_, C-3); 68.9 (CH, C-4); 151.4 (CH, C-4a); 102.1 (CH, C-5); 166.1 (C, C-6); 116.0 (C, C-7); 164.0 (C, C-8); 111.4 (C, C-8a); 53.1 (CH_2_, C-9); 56.7 (CH_3_, 6-OCH_3_); ESIMS *m*/*z* 236.9 [M − H]^−^.

#### 3.3.6. 6-Hydroxy-5,8-dimethoxy-3-methyl-1*H*-isochromen-1-one (**4**)

White powder; UV (MeOH) *λ*_max_ (log *ε*) 244 (2.76), 335 (2.07) nm; IR (neat) *ν*_max_ 3192, 2943, 2858, 1689, 1593, 1446, 1367, 1277, 1221, 1047, 980, 829, 746 cm^−1^; ^1^H NMR (400 MHz, MeOD) and ^13^C NMR (100 MHz, MeOD) data, see Table 1 and Table 2; HR-ESIMS *m*/*z* 237.0812 [M + H]^+^ (calculated for C_12_H_13_O_5_, 237.0758).

#### 3.3.7. (4*S*, 10*R*, 4’*S*)-Leptothalenone B (**5**)

Light brown amorphous powder; [${\left[\alpha \right]}_{D}^{20}$ = −11.9 (MeOH, *c* 0.21); UV (MeOH) *λ*_max_ (log *ε*) 222 (3.35), 286 (3.20), 326 (2.74) nm; CD (MeOH) *λ*_max_ (*Δε*) 227 (91.0), 289 (−149.5), 327 (29.1) nm; IR (neat) *ν*_max_ 3383, 2933, 1614, 1290, 1061, 831 cm^−1^; ^1^H NMR (400 MHz, MeOD) and ^13^C NMR (100 MHz, MeOD) data, see Table 1 and Table 2; HR-ESIMS *m/z* 495.1628 [M + Na]^+^ (calculated for C_25_H_28_O_9_Na, 495.1626).

#### 3.3.8. (4*R*, 10*S*, 4’*S*)-Leptothalenone B (**6**)

Light brown amorphous powder; [${\left[\alpha \right]}_{D}^{20}$ = 11.4 (MeOH, *c* 0.21); UV (MeOH) *λ*_max_ (log *ε*) 222 (3.35), 286 (3.20), 326 (2.74) nm; CD (MeOH) *λ*_max_ (*Δε*) 227 (11.5), 286 (−16.3), 315 (6.10) nm; IR (neat) *ν*_max_ 3383, 2933, 1614, 1290, 1061, 831 cm^−1^; ^1^H NMR (400 MHz, MeOD) and ^13^C NMR (100 MHz, MeOD) data, see Table 1 and Table 2; HR-ESIMS *m*/*z* 495.1628 [M + Na]^+^ (calculated for C_25_H_28_O_9_Na, 495.1626).

### 3.4. Cytotoxic Assay

All compounds were evaluated for their cytotoxicity against three human cancer cell lines, human lung adenocarcinoma (A549), human cervical carcinoma (HeLa), and the human breast adenocarcinoma cell line (MCF-7). The three tumor cell lines were generously provided by the cell bank of the Chinese Academy of Sciences (Shanghai, People’s Republic of China). The cytotoxic activities of the tested compounds were assayed according to the MTT method by using 96 well plates [13]. In brief, the cells were cultured in MEM medium, supplemented with 10% fetal bovine serum in a humidified atmosphere with 5% CO_2_ at 37 °C. Then, 198 μL adherent cells at the density of 4 × 10^4^ cell/mL were seeded into each well of the 96-well cell culture plates and incubated in 5% CO_2_ at 37 °C for 12 h to form a monolayer on the flat bottoms. Then, 2 μL test compounds dissolved in DMSO were added at concentrations of 50 μM. The plate was then incubated in 5% CO_2_ at 37 °C. After 24 h, the supernatant per well was removed and subsequently added to 90 μL fresh medium and 10 μL MTT was added into each well and incubated for 4 h. The supernatant per well was carefully removed, and 110 μL DMSO was added. The plate was then vortex shaken for 15 min to dissolve blue formazan crystals. The optical density (OD) of each well was measured on a microplate reader (Multiskan GO, Thermo Scientific) at a wavelength of 490 nm. Inhibition rate (%) = (OD_control_ − OD_treated_)/OD_control_ × 100%. 

### 3.5. Anti-Inflammation Bioassays

The anti-inflammation activity of the pure compounds was evaluated based on the reported procedures [14].

## 4. Conclusions

In summary, seven naphthalenones and one isocoumarine, including six new compounds, were isolated from the marine-derived fungus *Leptosphaerulina chartarum* 3608. The structures of the new compounds were established by analysis of HR-ESIMS and NMR spectroscopic data, and the absolute configurations were further determined by comparison of the experimental and calculated ECD spectra. Among all isolated compounds, only compound **6** showed moderate anti-inflammatory activity with IC_50_ = 44.5 ± 1.1 μM. All secondary metabolites **1**–**6** exhibited no cytotoxicity against A549, HeLa, and MCF-7 cancer cell lines at 50 μM.

## Figures and Tables

**Table 1:** ^1^H (400 MHz) NMR data of compounds **1**, **2**, and **4**–**6** (in CD_3_OD, *J* in Hz).

No.	1	2	4	5	6
2	2.62, m; 2.79, m	2.59, ddd (17.7, 10.1, 4.6); 2.77, ddd (17.7, 6.4, 4.6)		2.77, m; 2.60, m	2.77, m; 2.60, m
3	2.01, m; 2.26, m	2.01, m; 2.24, m		2.25, m; 2.00, m	2.25, m; 2.00, m
4	4.77, dd (8.9, 3.9)	4.76, dd (8.7, 3.9)	6.50, d (1.0)	4.73, ddd (13.2, 8.9, 3.9)	4.72, ddd (12.1, 9.1, 3.8)
5	6.79, s	6.75, s		6.66, s	6.61, s
7			6.50, s		
9	3.92, s	3.90, s	2.20, d (1.0)	3.83, s	3.79, s
10	5.31, q (6.8)	2.86, m	3.83, s	5.17, q (6.8)	5.17, q (6.8)
11	1.48, d (6.8)	3.56, t (7.5)	3.72, s	1.45, d (6.8)	1.44, d (6.8)
2′				2.77, m; 2.60, m	2.77, m; 2.60, m
3′				2.25, m; 2.00, m	2.25, m; 2.00, m
4′				4.73, ddd (13.2, 8.9, 3.9)	4.72, ddd (12.1, 9.1, 3.8)
5′				6.63, s	6.58, s
9′				3.79, s	3.76, s
10′				4.46, m	4.48, m

**Table 2:** ^13^C (100 MHz) NMR data of compounds **1**, **2**, and **4**–**6** (in CD_3_OD).

No.	1	2	4	5	6
1	204.6, C	203.0, C	162.1, C	204.4, C	204.4, C
2	36.1, CH_2_	34.5, CH_2_		36.1, CH_2_	36.1, CH_2_
3	32.7, CH_2_	31.4, CH_2_	156.3, C	32.7, CH_2_	32.8, CH_2_
4	68.6, CH	67.2, CH	98.9, CH	68.6, CH	68.7, CH
4a	150.2, C	147.7, C	135.3, C	151.3, C	151.2, C
5	102.2, CH	100.4, CH	135.8, C	101.9, CH	101.8, CH
6	164.8, C	164.1, C	158.5, C	166.2, C	166.1, C
7	119.1, C	111.9, C	100.2, CH	116.7, C	116.7, C
8	162.7, C	162.0, C	161.0, C	164.3, C	164.3, C
8a	111.1, C	109.6, C	101.2, C	110.8, C	110.8, C
9	56.4, CH_3_	55.0, CH_3_	19.5, CH_3_	56.3, CH_3_	56.2, CH_3_
10	63.4, CH	25.3, CH_2_	56.4, CH_3_	70.3, CH	70.4, CH
11	22.2, CH_3_	60.1, CH_2_	61.6, CH_3_	20.0, CH_3_	20.1, CH_3_
1′				204.2, C	204.2, C
2′				36.1, CH_2_	36.1, CH_2_
3’				32.7, CH_2_	32.8, CH_2_
4′				68.6, CH	68.6, CH
4a′				150.3, C	150.3, C
5′				101.3, CH	101.3, CH
6′				165.9, C	165.9, C
7′				113.0, C	113.1, C
8′				163.7, C	163.6, C
8a′				110.7, C	110.7, C
9′				56.2, CH_3_	56.1, CH_3_
10′				59.6, CH_2_	59.8, CH_2_

## Supplementary Materials

The following are available online at http://www.mdpi.com/1660-3397/16/5/173/s1. HR-ESIMS, NMR, CD spectra of the new compounds as well as other supporting data.
