# Supplementary material for: Mono- and Dimeric Naphthalenones from the Marine-Derived Fungus Leptosphaerulina chartarum 3608
Source: Mar Drugs. 2018 May 21;16(5):173. doi: 10.3390/md16050173 (PMC5983304; doi:10.3390/md16050173)

Supplementary materials for

Mono- and dimeric naphthalenones from marine-derived fungus *Leptosphaerulina chartarum* 3608

Panpan Zhang [1], Chunxiu Jia [2], Jiajia Lang [2], Jing Li [1], Guangyuan Luo [1], Senhua Chen [1,*], Sujun Yan [2,*], and Lan Liu [1,3]

[1] School of Marine Sciences, Sun Yat-sen University, Guangzhou 510006, People's Republic of China; zhangpp23@mail2.sysu.edu.cn; Lijing356@mail.sysu.edu.cn; luogy331@gmail.com; cesllan@mail.sysu.edu.cn

[2] School of Chemistry, Sun Yat-sen University, Guangzhou 510006, People's Republic of China; jiachx@mail2.sysu.edu.cn; langjj@mail2.sysu.edu.cn

[3] Key Laboratory of Functional Molecules from Oceanic Microorganisms, Sun Yat-sen University, Department of Education of Guangdong Province, Guangzhou 510080, China

***** Correspondence: chensenh@mail.sysu.edu.cn; cesyansj@mail.sysu.edu.cn; Tel.: +86-20-84725459

Content

Figure S1. ^{1}H (400 MHz) NMR spectrum of (±)-leptothalenone A (**1**) in CD_3OD. 4

Figure S2. ^{13}C (100 MHz) NMR spectrum of (±)-leptothalenone A (**1**) in CD_3OD. 4

Figure S3. DEPT-90 spectrum of (±)-leptothalenone A (**1**) in CD_3OD. 5

Figure S4. DEPT-135 spectrum of (±)-leptothalenone A (**1**) in CD_3OD. 5

Figure S5. HSQC spectrum of (±)-leptothalenone A (**1**) in CD_3OD. 6

Figure S6. HMBC spectrum of (±)-leptothalenone A (**1**) in CD_3OD. 6

Figure S7. ^{1}H-^{1}H COSY spectrum of (±)-leptothalenone A (**1**) in CD_3OD. 7

Figure S8. NOESY spectrum of (±)-leptothalenone A (**1**) in CD_3OD. 7

Figure S9. HR-ESIMS of (±)-leptothalenone A (**1**) in CD_3OD. 8

Figure S10. ^{1}H (400 MHz) NMR spectrum of (-)-4,8-dihydroxy-7- (2-hydroxy-ethyl)-6-methoxy-3,4-dihydro-2*H*-naphthalen-1-one ((-)-**2**) in CD_3OD. 8

Figure S11. ^{13}C (100 MHz) NMR spectrum of (-)-4,8-dihydroxy-7- (2-hydroxy-ethyl)-6-methoxy-3,4-dihydro-2*H*-naphthalen-1-one ((-)-**2**) in CD_3OD. 9

Figure S12. DEPT-90 spectrum of (-)-4,8-dihydroxy-7- (2-hydroxy-ethyl)-6-methoxy-3,4-dihydro-2*H*-naphthalen-1-one ((-)-**2**) in CD_3OD. 9

Figure S13. DEPT-135 spectrum of (-)-4,8-dihydroxy-7- (2-hydroxy-ethyl)-6-methoxy-3,4-dihydro-2*H*-naphthalen-1-one ((-)-**2**) in CD_3OD. 10

Figure S14. HSQC spectrum of (-)-4,8-dihydroxy-7- (2-hydroxy-ethyl)-6-methoxy-3,4-dihydro-2*H*-naphthalen-1-one ((-)-**2**) in CD_3OD. 10

Figure S15. HMBC spectrum of (-)-4,8-dihydroxy-7- (2-hydroxy-ethyl)-6-methoxy-3,4-dihydro-2*H*-naphthalen-1-one ((-)-**2**) in CD_3OD. 11

Figure S16. ^{1}H-^{1}H COSY spectrum of (-)-4,8-dihydroxy-7- (2-hydroxy-ethyl)-6-methoxy-3,4-dihydro-2*H*-naphthalen-1-one ((-)-**2**) in CD_3OD. 11

Figure S17. NOESY spectrum of (-)-4,8-dihydroxy-7- (2-hydroxy-ethyl)-6-methoxy-3,4-dihydro-2*H*-naphthalen-1-one ((-)-**2**) in CD_3OD. 12

Figure S18. HR-ESIMS of (-)-4,8-dihydroxy-7- (2-hydroxy-ethyl)-6-methoxy-3,4-dihydro-2*H*-naphthalen-1-one ((-)-**2**) in CD_3OD. 12

Figure S19. ^{1}H (400 MHz) NMR spectrum of 6-hydroxy-5,8-dimethoxy-3-methyl-1*H*-isochromen-1-one (**4**) in CD_3OD. 13

Figure S20. ^{13}C (100 MHz) NMR spectrum of 6-hydroxy-5,8-dimethoxy-3-methyl-1*H*-isochromen-1-one (**4**) in CD_3OD. 13

Figure S21. DEPT-90 spectrum of 6-hydroxy-5,8-dimethoxy-3-methyl-1*H*-isochromen-1-one (**4**) in CD_3OD. 14

Figure S22. DEPT-135 spectrum of 6-hydroxy-5,8-dimethoxy-3-methyl-1*H*-isochromen-1-one (**4**) in CD_3OD. 14

Figure S23. HSQC spectrum of 6-hydroxy-5,8-dimethoxy-3-methyl-1*H*-isochromen-1-one (**4**) in CD_3OD. 15

Figure S23. HMBC spectrum of 6-hydroxy-5,8-dimethoxy-3-methyl-1*H*-isochromen-1-one (**4**) in CD_3OD. 15

Figure S25 ^1H-^1H COSY spectrum of 6-hydroxy-5,8-dimethoxy-3-methyl-1*H*-isochromen-1-one (**4**) in CD_3OD. 16

Figure S26. HR-ESIMS of 6-hydroxy-5,8-dimethoxy-3-methyl-1*H*-isochromen-1-one (**4**) in CD_3OD. 16

Figure S27. ^1H (400 MHz) NMR spectrum of (4*S*, 10*R*, 4'*S*)-leptothalenone B (**5**) in CD_3OD. 17

Figure S28. ^{13}C (100 MHz) NMR spectrum of (4*S*, 10*R*, 4'*S*)-leptothalenone B (**5**) in CD_3OD. 17

Figure S29. DEPT-90 spectrum of (4*S*, 10*R*, 4'*S*)-leptothalenone B (**5**) in CD_3OD. 18

Figure S30. DEPT-135 spectrum of (4*S*, 10*R*, 4'*S*)-leptothalenone B (**5**) in CD_3OD. 18

Figure S31. HSQC spectrum of (4*S*, 10*R*, 4'*S*)-leptothalenone B (**5**) in CD_3OD. 19

Figure S32. HMBC spectrum of (4*S*, 10*R*, 4'*S*)-leptothalenone B (**5**)in CD_3OD. 19

Figure S33. ^1H-^1H COSY spectrum of (4*S*, 10*R*, 4'*S*)-leptothalenone B (**5**) in CD_3OD. 20

Figure S34. NOESY spectrum of (4*S*, 10*R*, 4'*S*)-leptothalenone B (**5**) in CD_3OD. 20

Figure S35. HR-ESIMS of (4*S*, 10*R*, 4'*S*)-leptothalenone B (**5**). 21

Figure S36. ^1H (400 MHz) NMR spectrum of (4*R*, 10*S*, 4'*S*)-leptothalenone B (**6**) in CD_3OD. 21

Figure S37. ^{13}C (100 MHz) NMR spectrum of (4*R*, 10*S*, 4'*S*)-leptothalenone B (**6**) in CD_3OD. 22

Figure S38. DEPT-90 spectrum of (4*R*, 10*S*, 4'*S*)-leptothalenone B (**6**) in CD_3OD. 22

Figure S39. DEPT-135 spectrum of (4*R*, 10*S*, 4'*S*)-leptothalenone B (**6**) in CD3OD. 23

Figure S40. HSQC spectrum of (4*R*, 10*S*, 4'*S*)-leptothalenone B (**6**) in CD_3OD. 23

Figure S41. HMBC spectrum of (4*R*, 10*S*, 4'*S*)-leptothalenone B (**6**) in CD_3OD. 24

Figure S42. ^1H-^1H COSY spectrum of (4*R*, 10*S*, 4'*S*)-leptothalenone B (**6**) in CD_3OD. 24

Figure S43. NOESY spectrum of (4*R*, 10*S*, 4'*S*)-leptothalenone B (**6**) in CD_3OD. 25

Figure S44. HR-ESIMS of (4*R*, 10*S*, 4'*S*)-leptothalenone B (**6**) in CD_3OD. 25

Figure S45. Structure of **3** resulting from single-crystal X-ray diffraction. 26

Figure S1. ^{1}H (400 MHz) NMR spectrum of (±)-leptothalenone A (**1**) in CD_3OD.

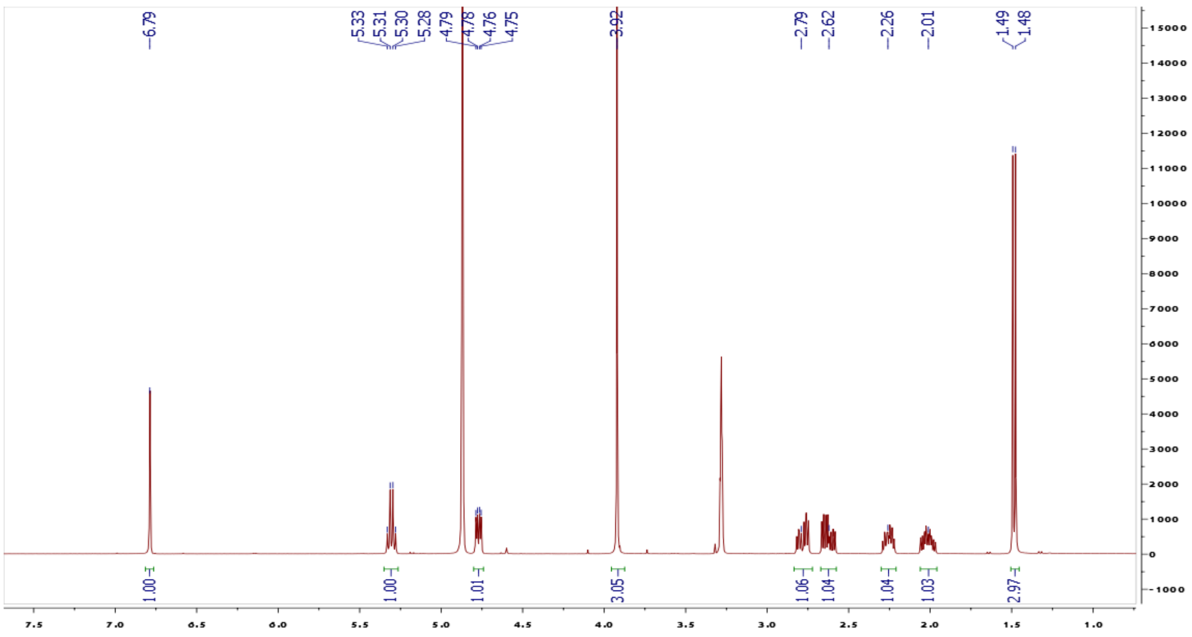

Figure S2. ^{13}C (100 MHz) NMR spectrum of (±)-leptothalenone A (**1**) in CD_3OD.

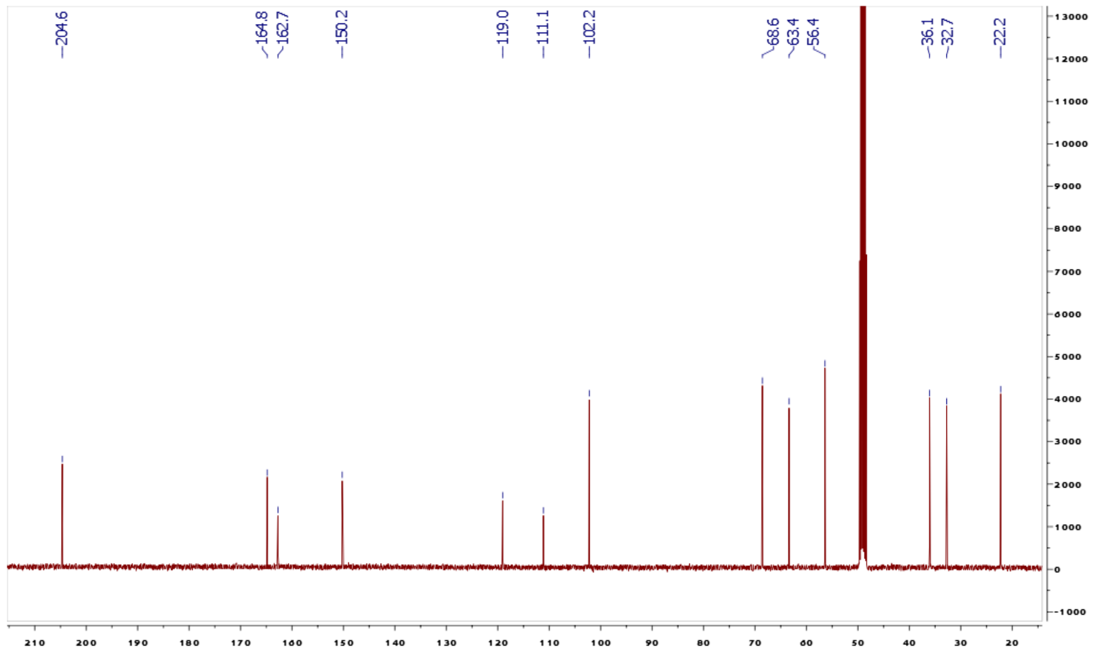

Figure S3. DEPT-90 spectrum of (±)-leptothalenone A (**1**) in CD_3OD.

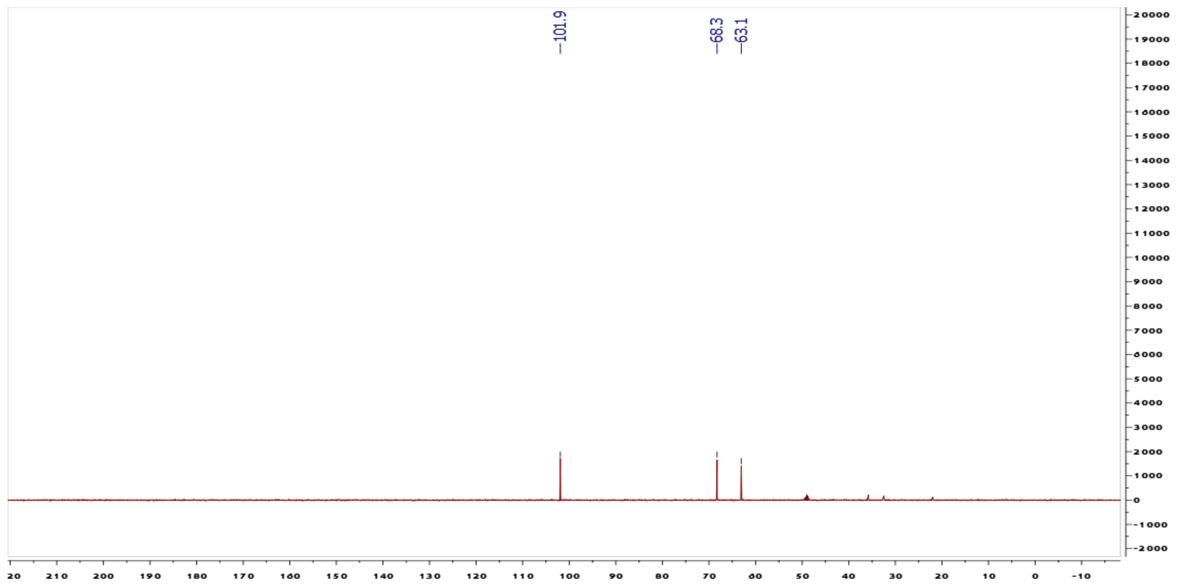

Figure S4. DEPT-135 spectrum of (±)-leptothalenone A (**1**) in CD_3OD.

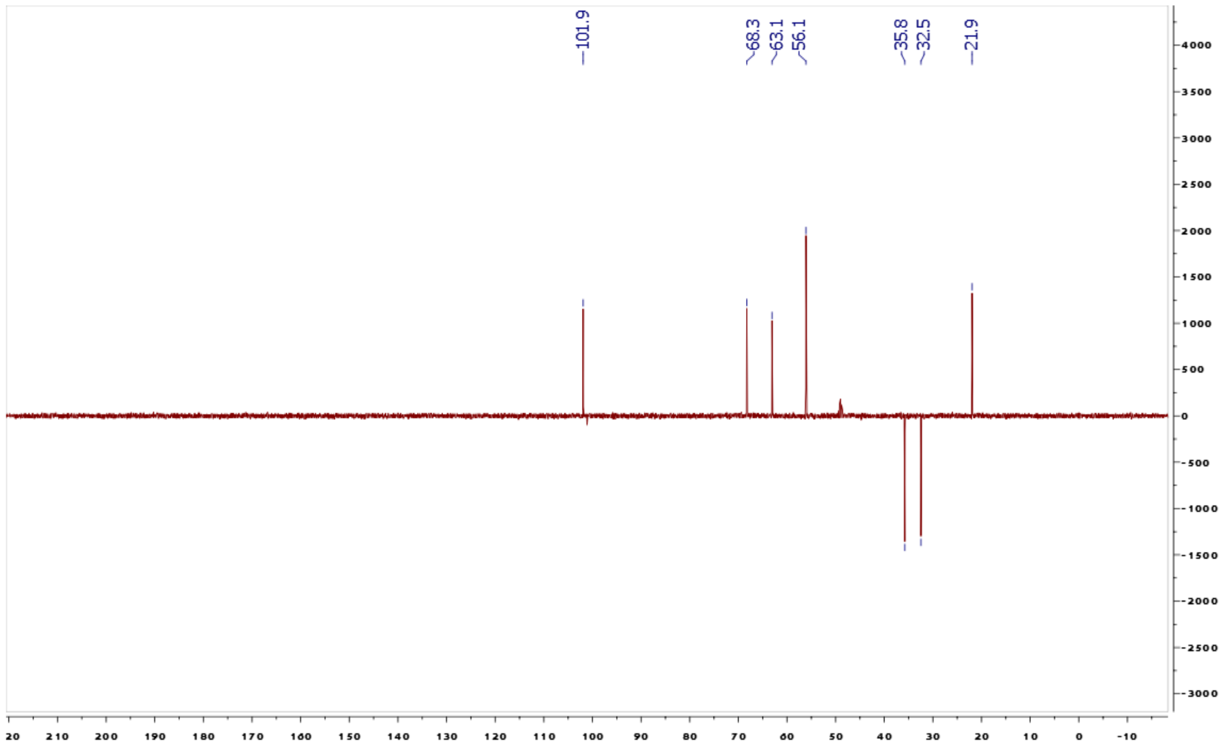

Figure S5. HSQC spectrum of (±)-leptothalenone A (**1**) in CD_3OD.

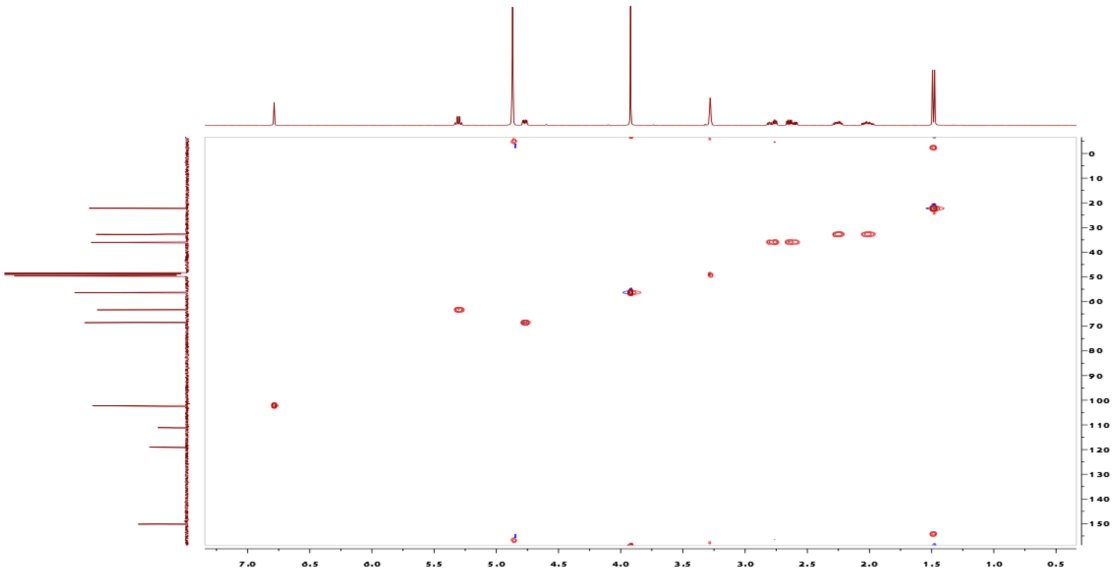

Figure S6. HMBC spectrum of (±)-leptothalenone A (**1**) in CD_3OD.

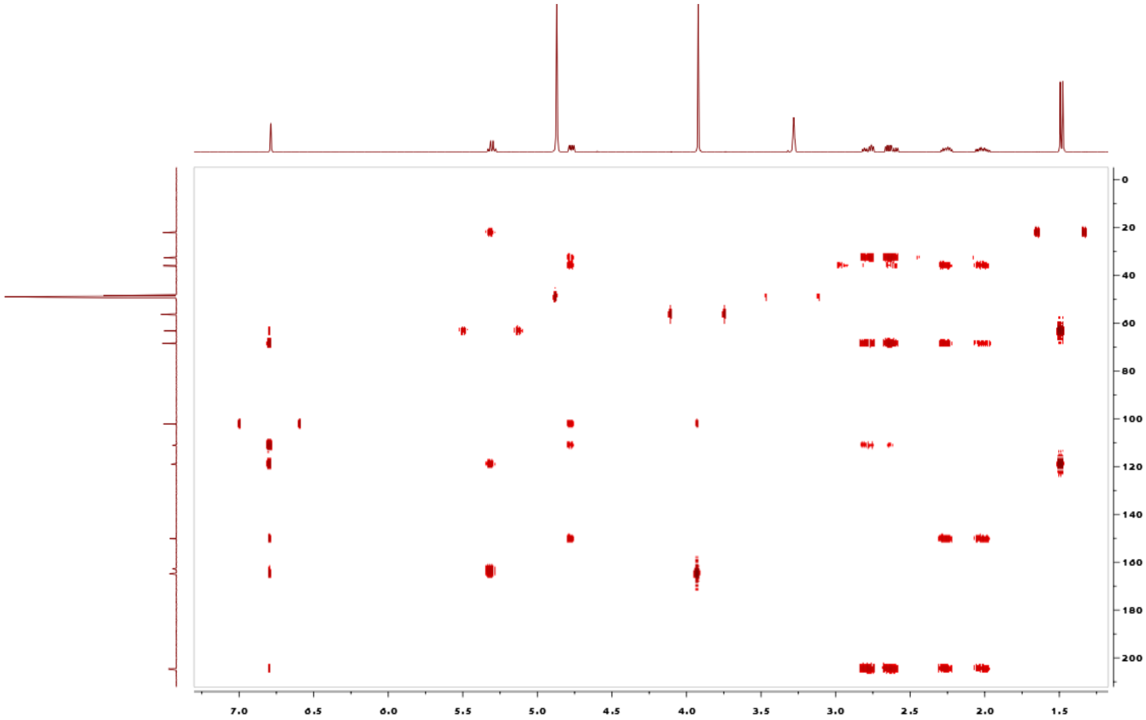

Figure S7. ^{1}H-^{1}H COSY spectrum of (±)-leptothalenone A (**1**) in CD_3OD.

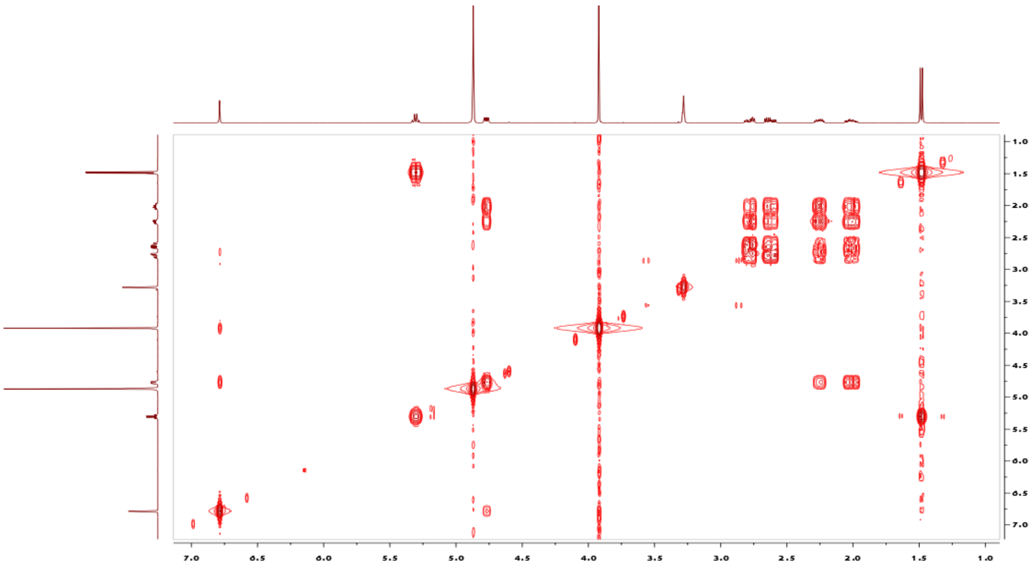

Figure S8. NOESY spectrum of (±)-leptothalenone A (**1**) in CD_3OD.

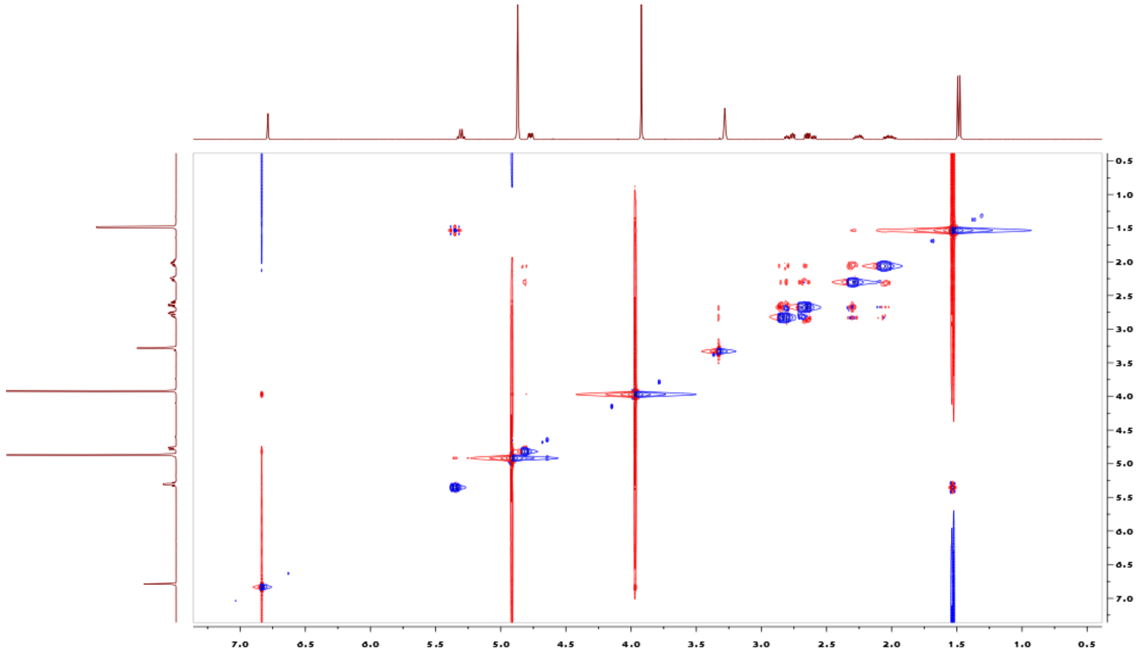

Figure S9. HR-ESIMS of (±)-leptothalenone A (**1**) in CD_3OD.

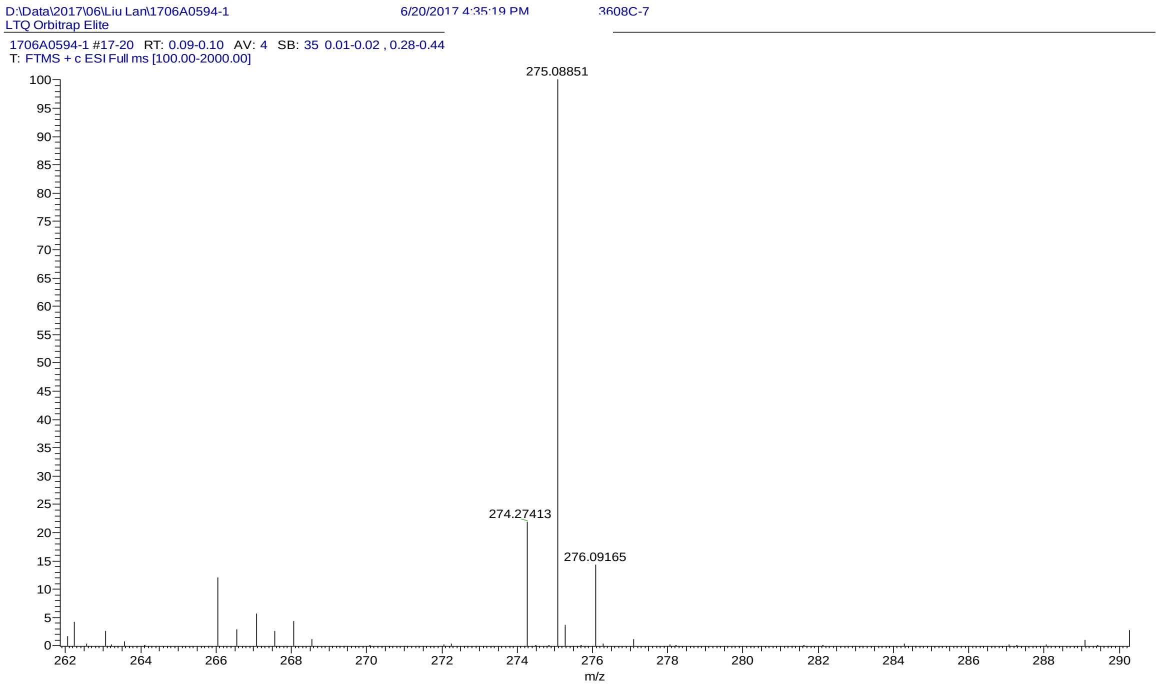

SPECTRUM - simulation :

m/z	Theo. Mass	Delta (ppm)	RDB equiv.	Composition
275.08865	275.08899	-1.25	5.5	$C_{13}H_{16}O_5Na$

Figure S10. ^{1}H (400 MHz) NMR spectrum of (-)-4,8-dihydroxy-7- (2-hydroxy-ethyl)-6-methoxy-3,4-dihydro-2*H*-naphthalen-1-one ((-)-**2**) in CD_3OD.

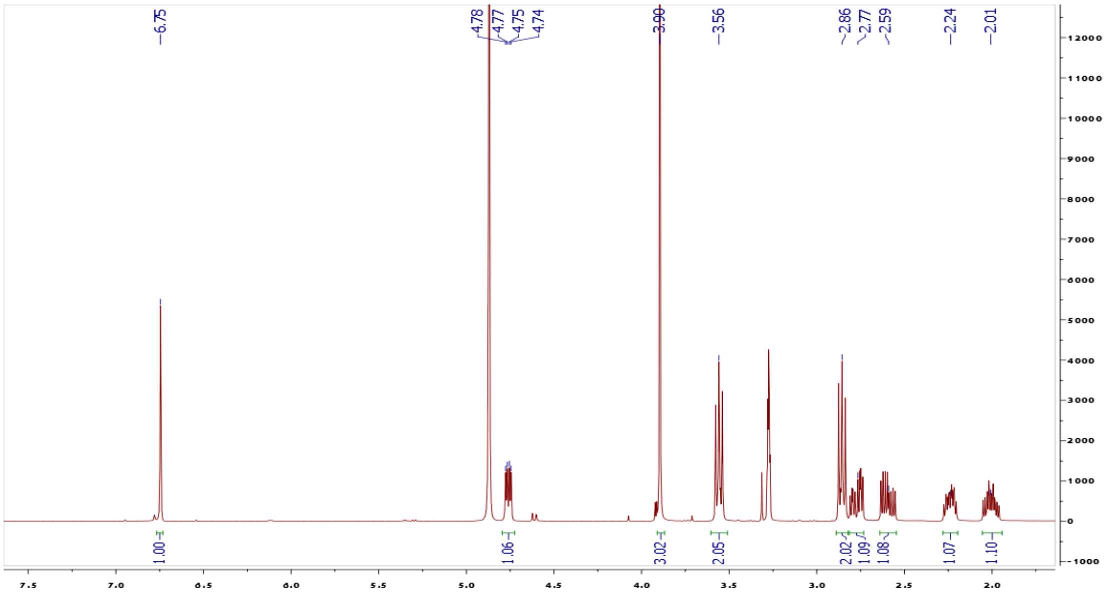

Figure S11. ^{13}C (100 MHz) NMR spectrum of (-)-4,8-dihydroxy-7- (2-hydroxy-ethyl)-6-methoxy-3,4-dihydro-2*H*-naphthalen-1-one ((-)-**2**) in CD_3OD.

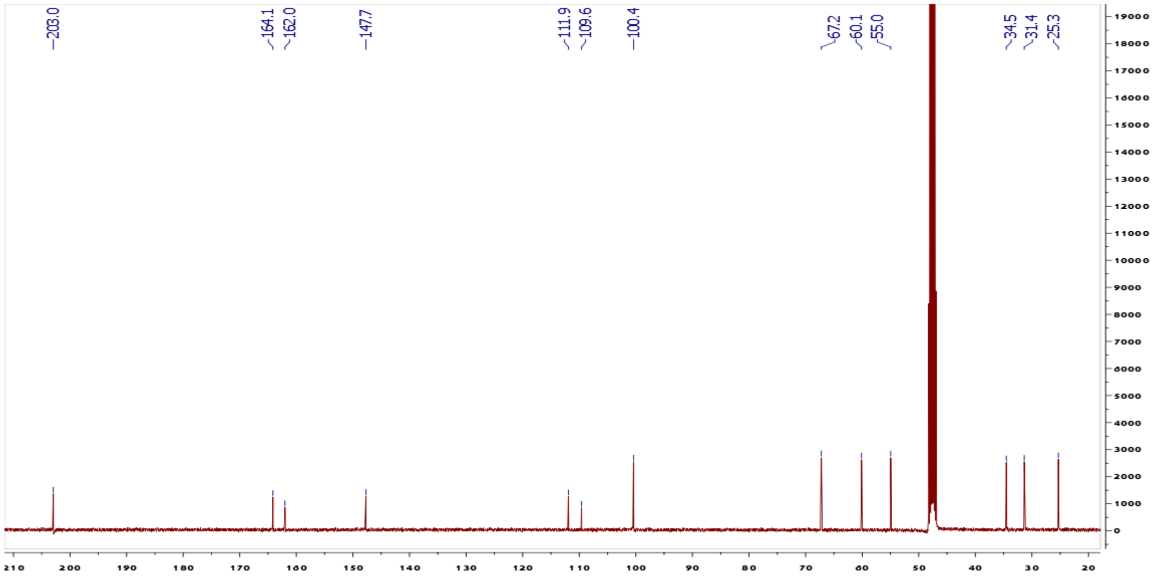

Figure S12. DEPT-90 spectrum of (-)-4,8-dihydroxy-7- (2-hydroxy-ethyl)-6-methoxy-3,4-dihydro-2*H*-naphthalen-1-one ((-)-**2**) in CD_3OD.

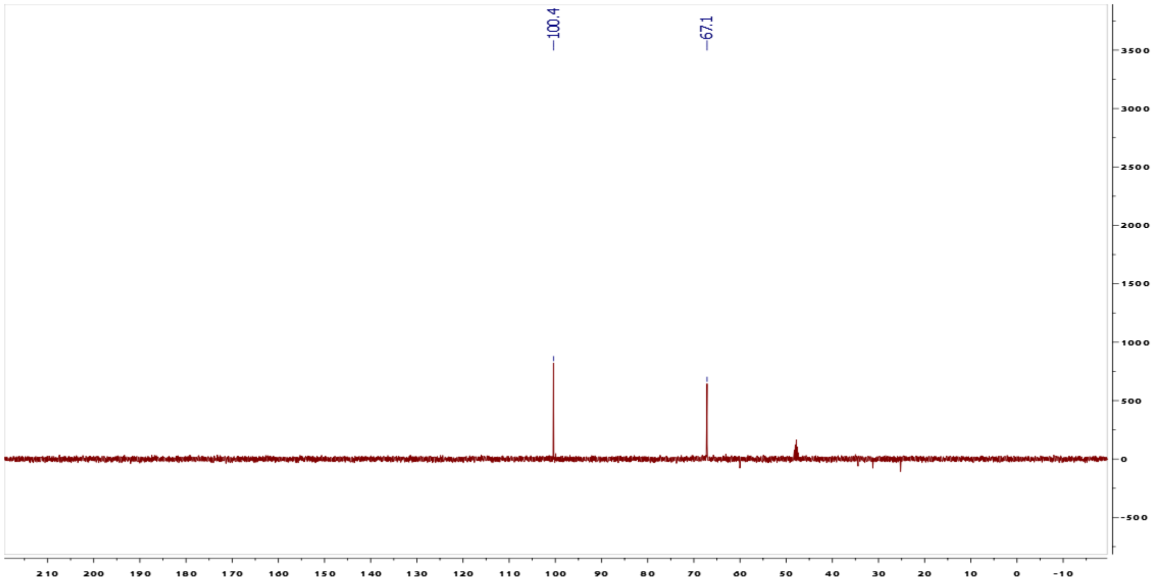

Figure S13. DEPT-135 spectrum of (-)-4,8-dihydroxy-7- (2-hydroxy-ethyl)-6-methoxy-3,4-dihydro-2*H*-naphthalen-1-one ((-)-**2**) in CD_3OD.

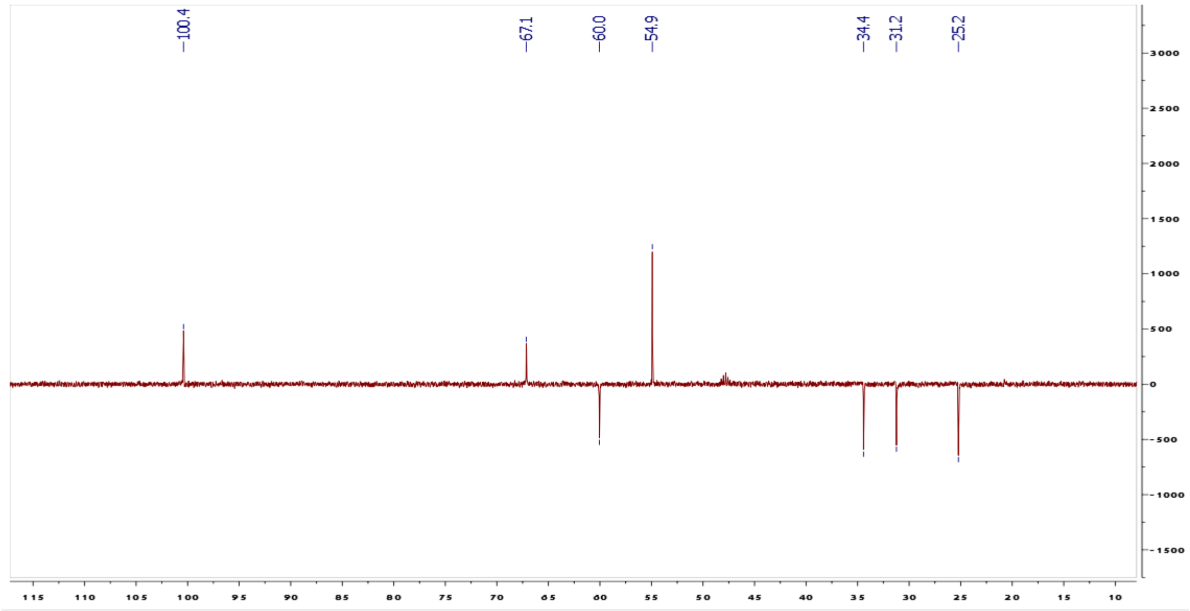

Figure S14. HSQC spectrum of (-)-4,8-dihydroxy-7- (2-hydroxy-ethyl)-6-methoxy-3,4-dihydro-2*H*-naphthalen-1-one ((-)-**2**) in CD_3OD.

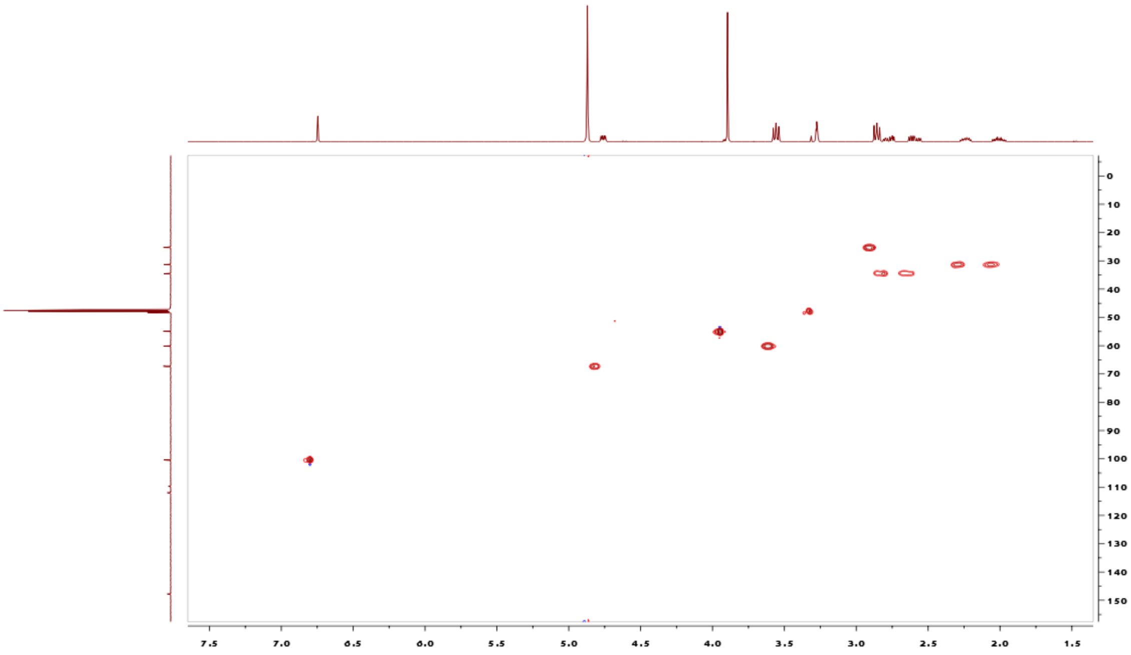

Figure S15. HMBC spectrum of (-)-4,8-dihydroxy-7- (2-hydroxy-ethyl)-6-methoxy-3,4-dihydro-2*H*-naphthalen-1-one ((-)-**2**) in CD_3OD.

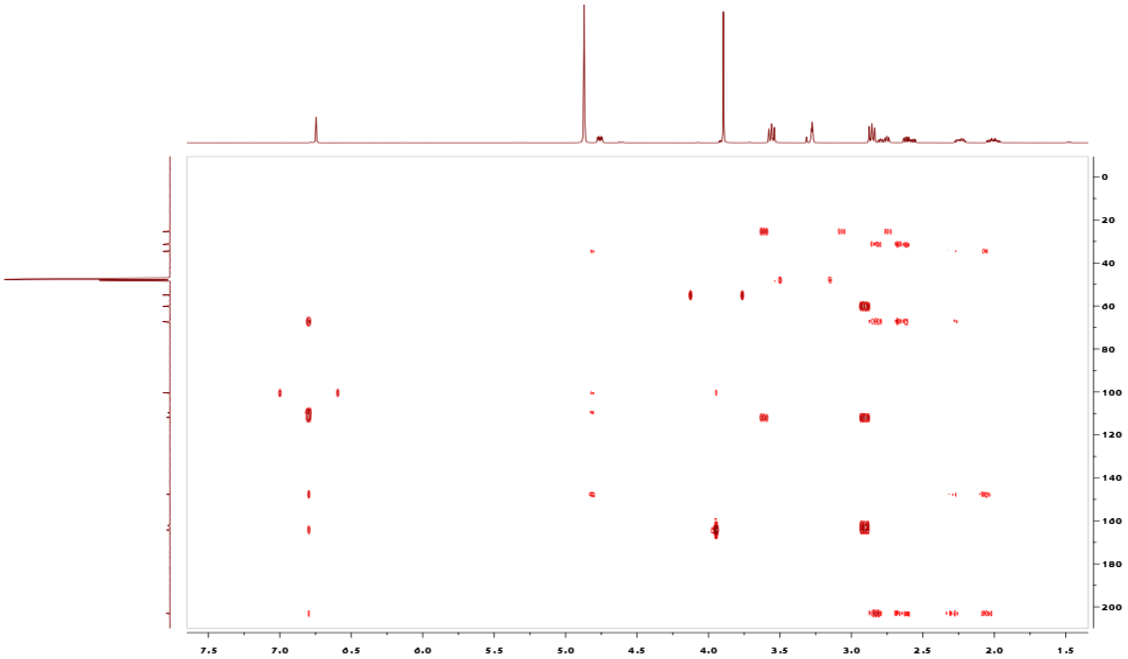

Figure S16. ^1H-^1H COSY spectrum of (-)-4,8-dihydroxy-7- (2-hydroxy-ethyl)-6-methoxy-3,4-dihydro-2*H*-naphthalen-1-one ((-)-**2**) in CD_3OD.

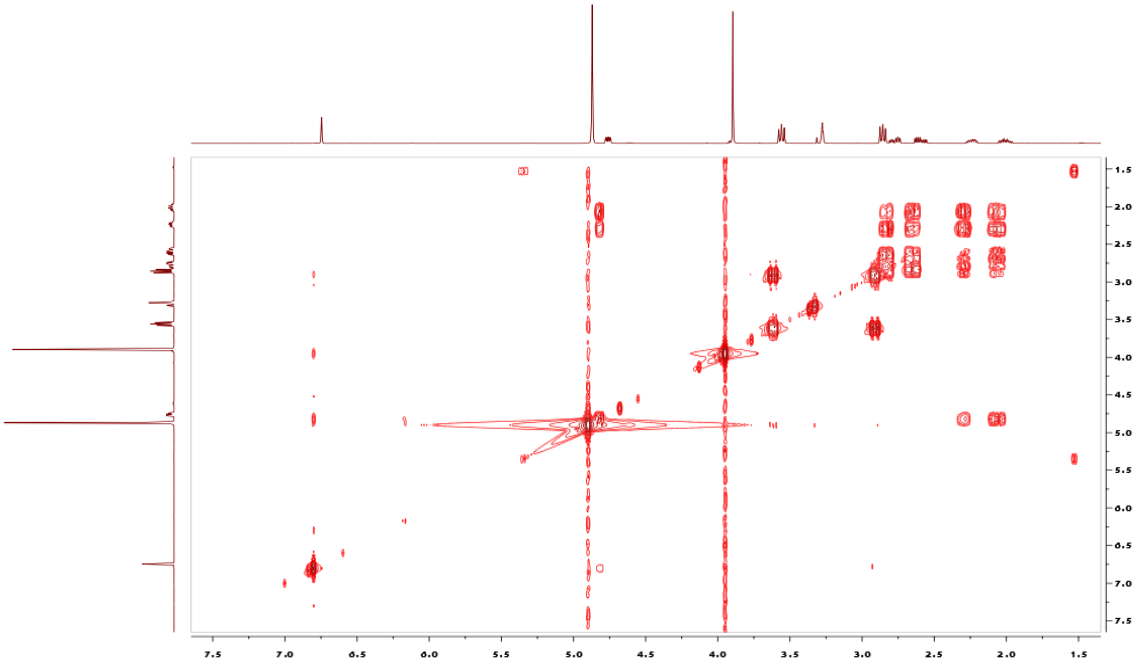

Figure S17. NOESY spectrum of (-)-4,8-dihydroxy-7- (2-hydroxy-ethyl)-6-methoxy-3,4-dihydro-2*H*-naphthalen-1-one ((-)-**2**) in CD_3OD.

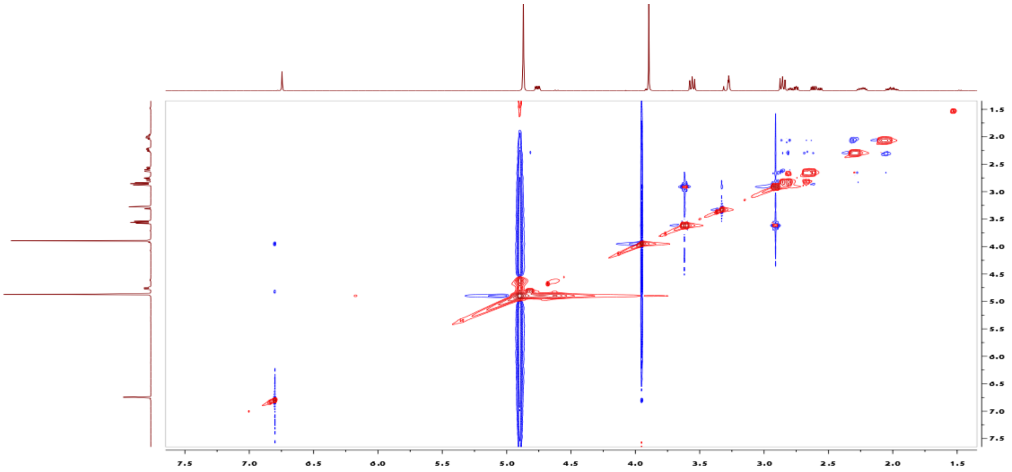

Figure S18. ESIMS of (-)-4,8-dihydroxy-7- (2-hydroxy-ethyl)-6-methoxy-3,4-dihydro-2*H*-naphthalen-1-one ((-)-**2**) in CD_3OD.

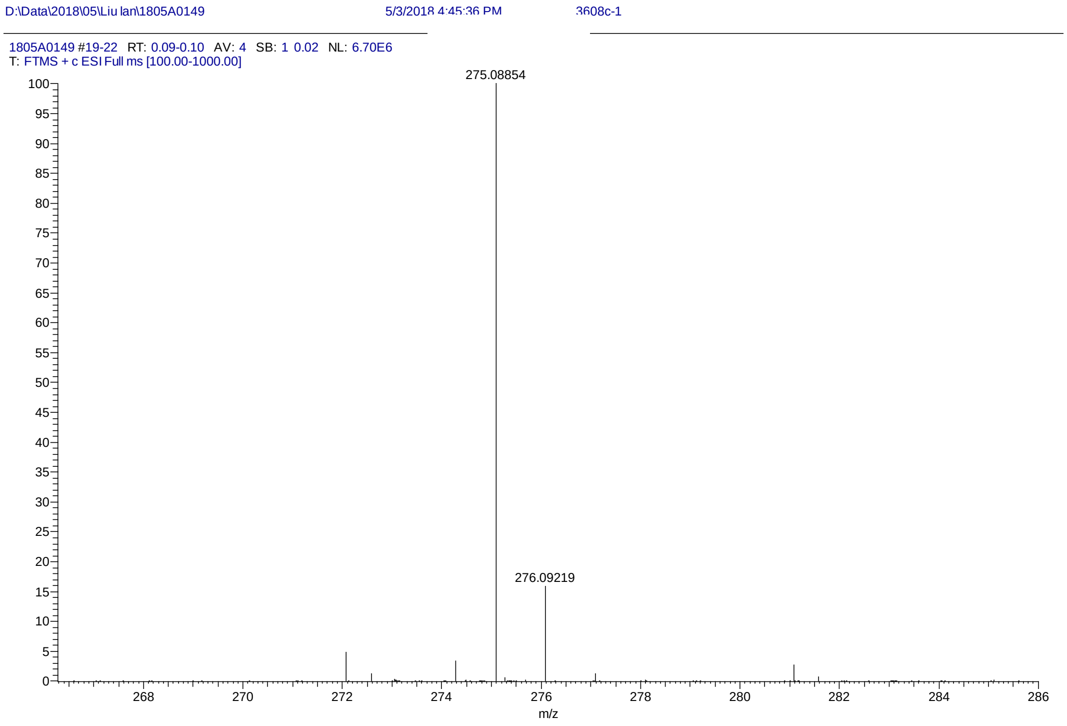

SPECTRUM - simulation :

m/z	Theo. Mass	Delta (ppm)	RDB equiv.	Composition
275.08854	275.08899	-1.65	5.5	$C_{13}H_{16}O_5Na$

Figure S19. ^{1}H (400 MHz) NMR spectrum of 6-hydroxy-5,8-dimethoxy-3-methyl-1*H*-isochromen-1-one (**4**) in CD_3OD.

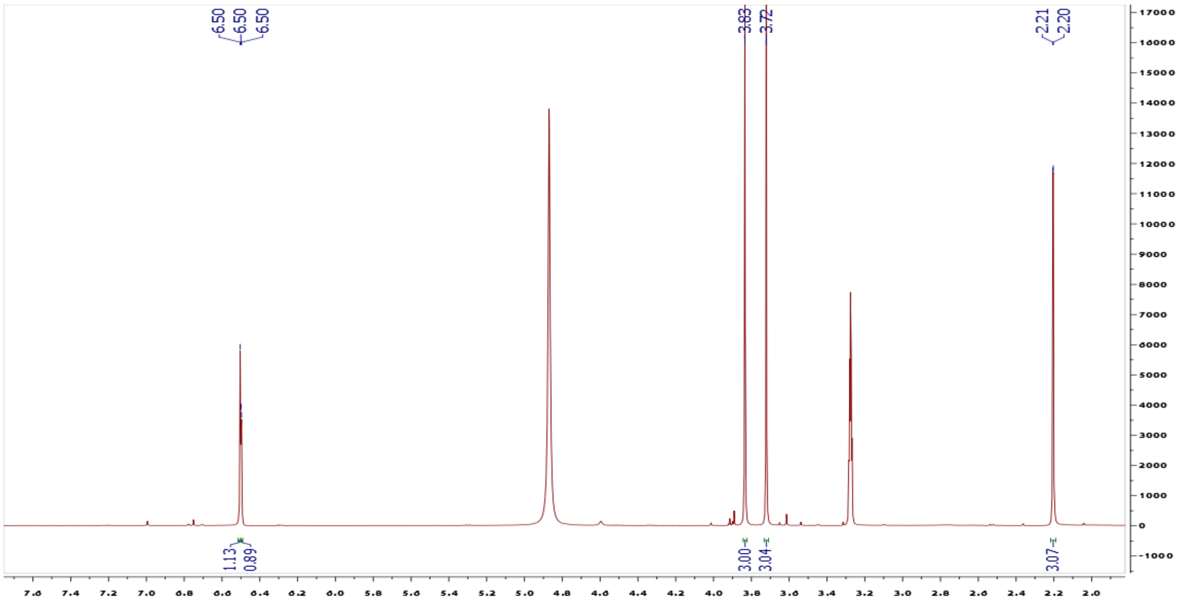

Figure S20. ^{13}C (100 MHz) NMR spectrum of 6-hydroxy-5,8-dimethoxy-3-methyl-1*H*-isochromen-1-one (**4**) in CD_3OD.

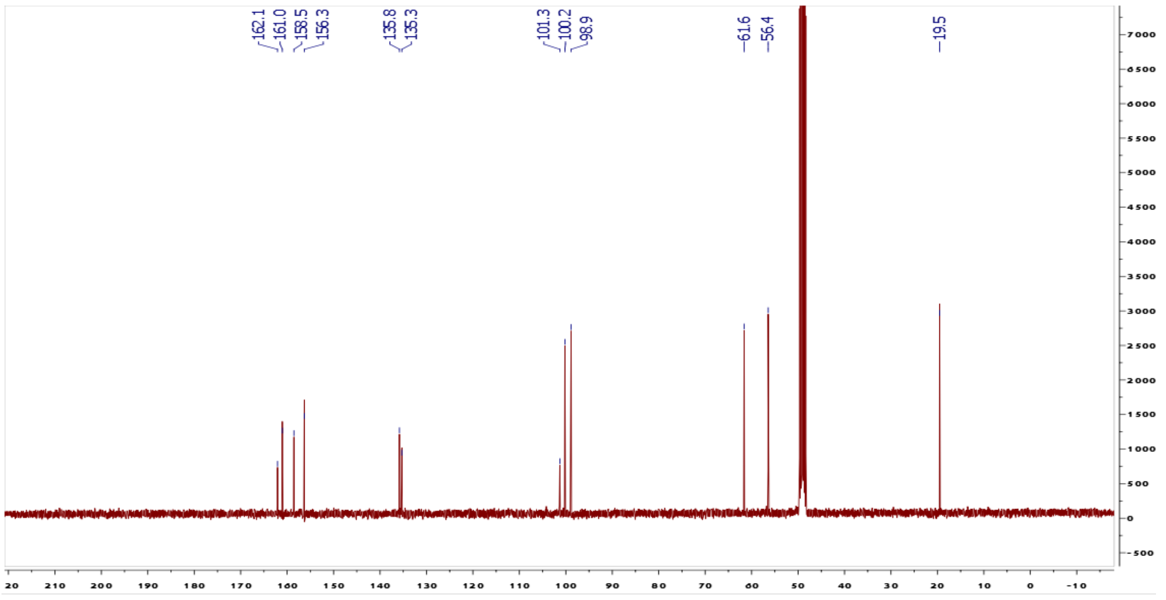

Figure S21. DEPT-90 spectrum of 6-hydroxy-5,8-dimethoxy-3-methyl-1*H*-isochromen-1-one (**4**) in CD_3OD.

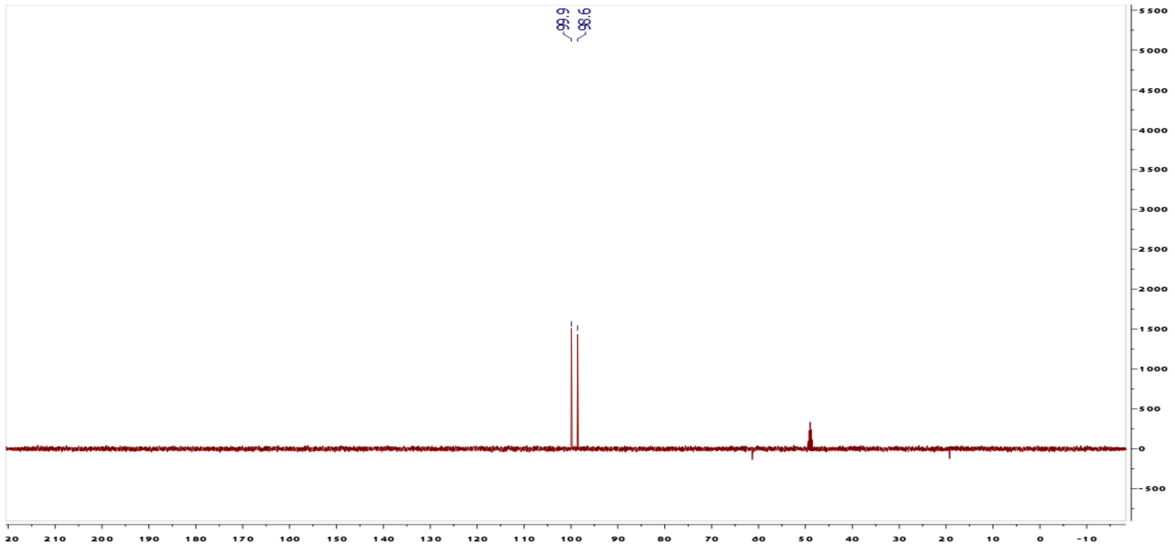

Figure S22. DEPT-135 spectrum of 6-hydroxy-5,8-dimethoxy-3-methyl-1*H*-isochromen-1-one (**4**) in CD_3OD.

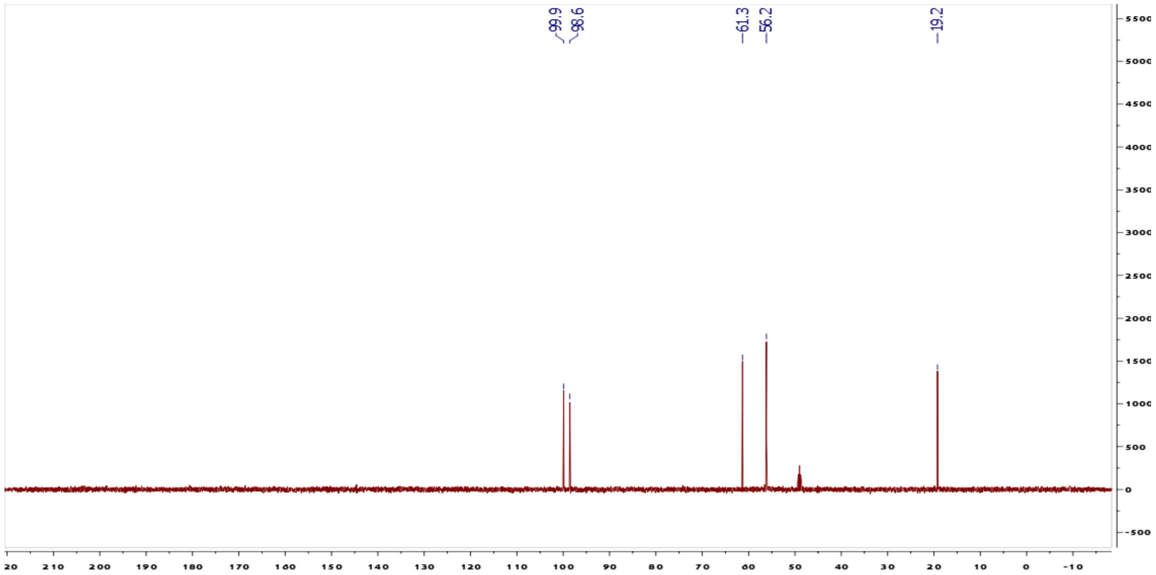

Figure S23. HSQC spectrum of 6-hydroxy-5,8-dimethoxy-3-methyl-1*H*-isochromen-1-one (**4**) in CD_3OD.

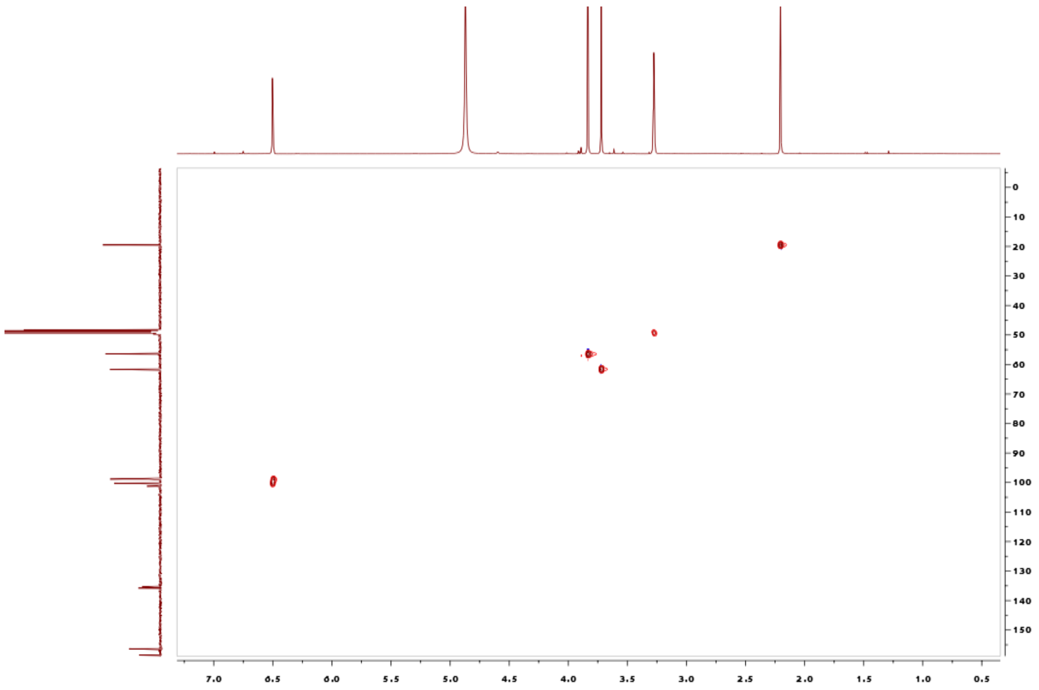

Figure S23. HMBC spectrum of 6-hydroxy-5,8-dimethoxy-3-methyl-1*H*-isochromen-1-one (**4**) in CD_3OD.

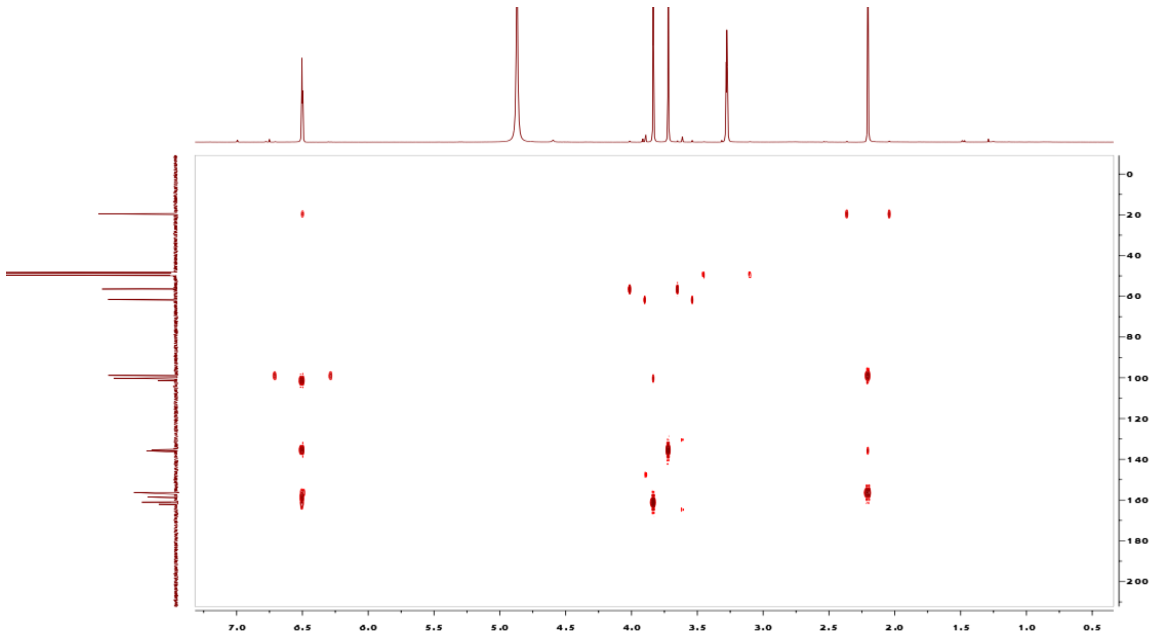

Figure S25 ^{1}H-^{1}H COSY spectrum of 6-hydroxy-5,8-dimethoxy-3-methyl-1*H*-isochromen-1-one (**4**) in CD_3OD.

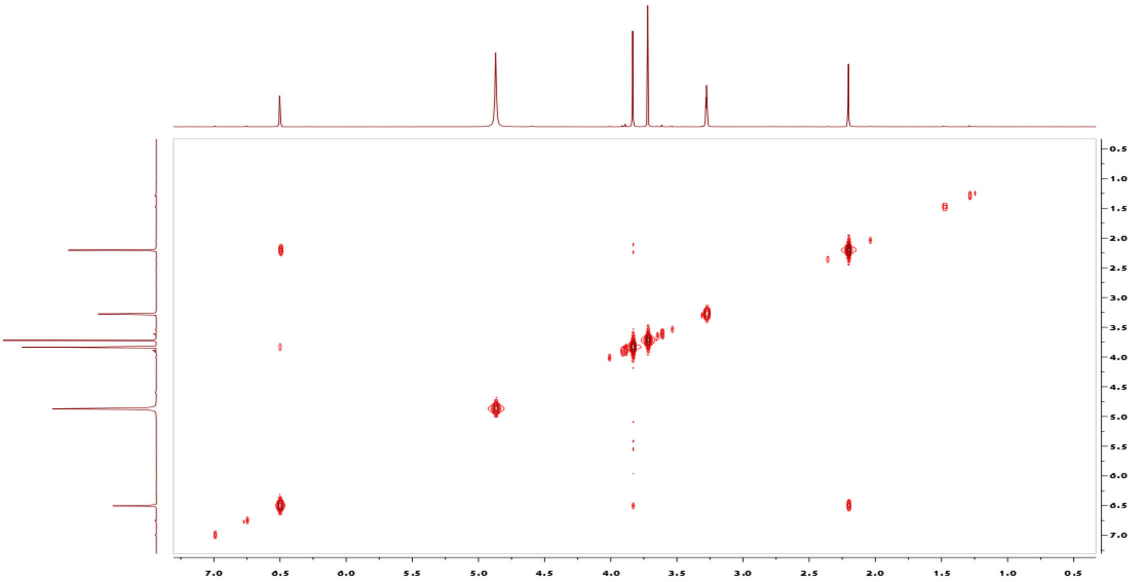

Figure S26. HR-ESIMS of 6-hydroxy-5,8-dimethoxy-3-methyl-1*H*-isochromen-1-one (**4**) in CD_3OD.

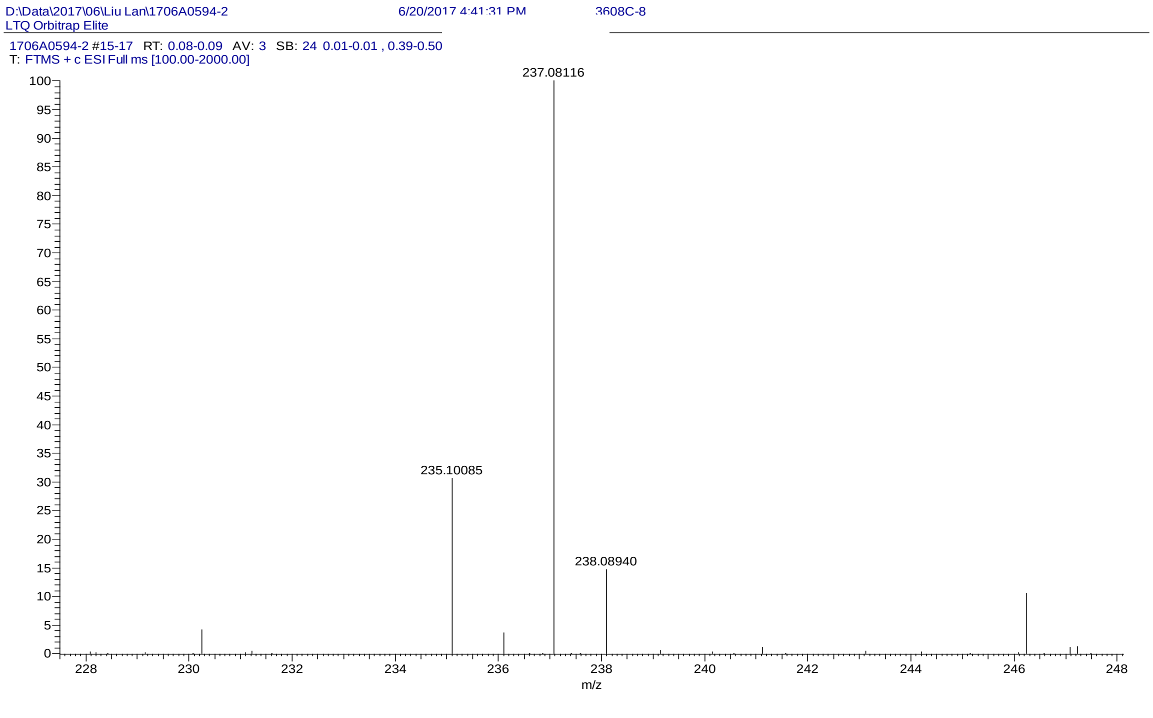

SPECTRUM - simulation :

m/z	Theo. Mass	Delta (ppm)	RDB equiv.	Composition
237.07572	237.07575	-0.13	6.5	$C_{12}H_{13}O_5$

Figure S27. ^{1}H (400 MHz) NMR spectrum of (4*S*, 10*R*, 4'*S*)-leptothalenone B (**5**) in CD_3OD.

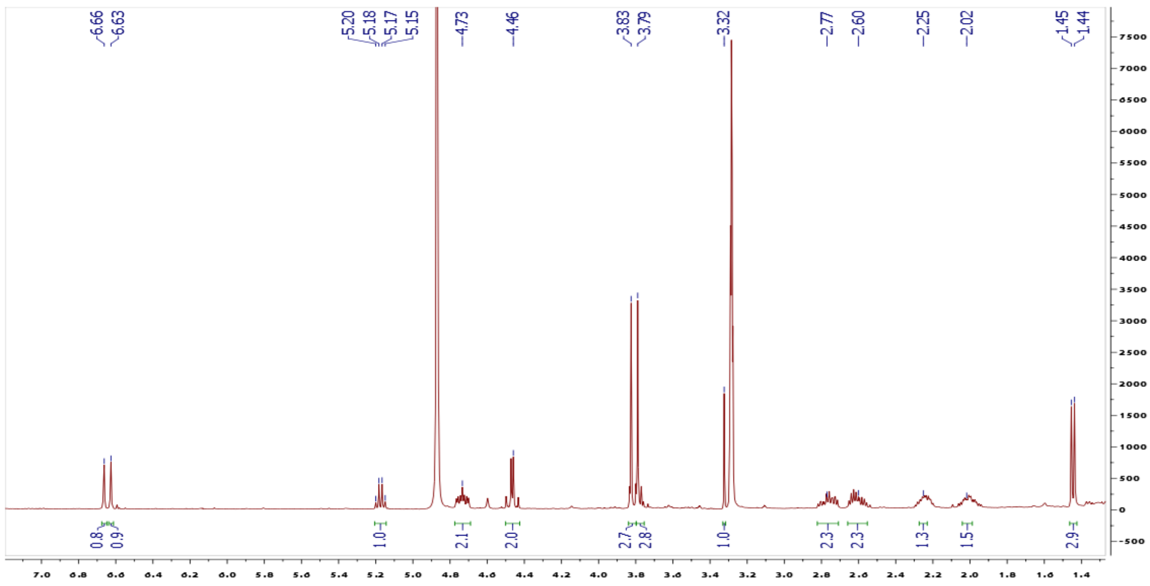

Figure S28. ^{13}C (100 MHz) NMR spectrum of (4*S*, 10*R*, 4'*S*)-leptothalenone B (**5**) in CD_3OD.

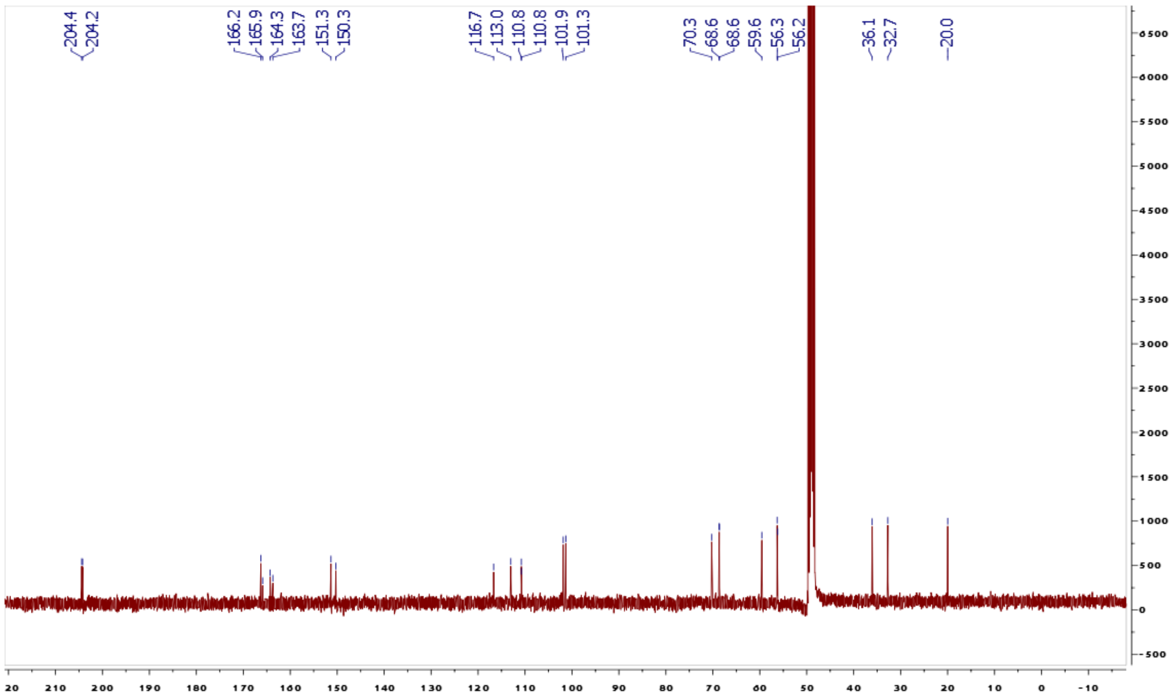

Figure S29. DEPT-90 spectrum of (4*S*, 10*R*, 4'*S*)-leptothalenone B (**5**) in CD_3OD.

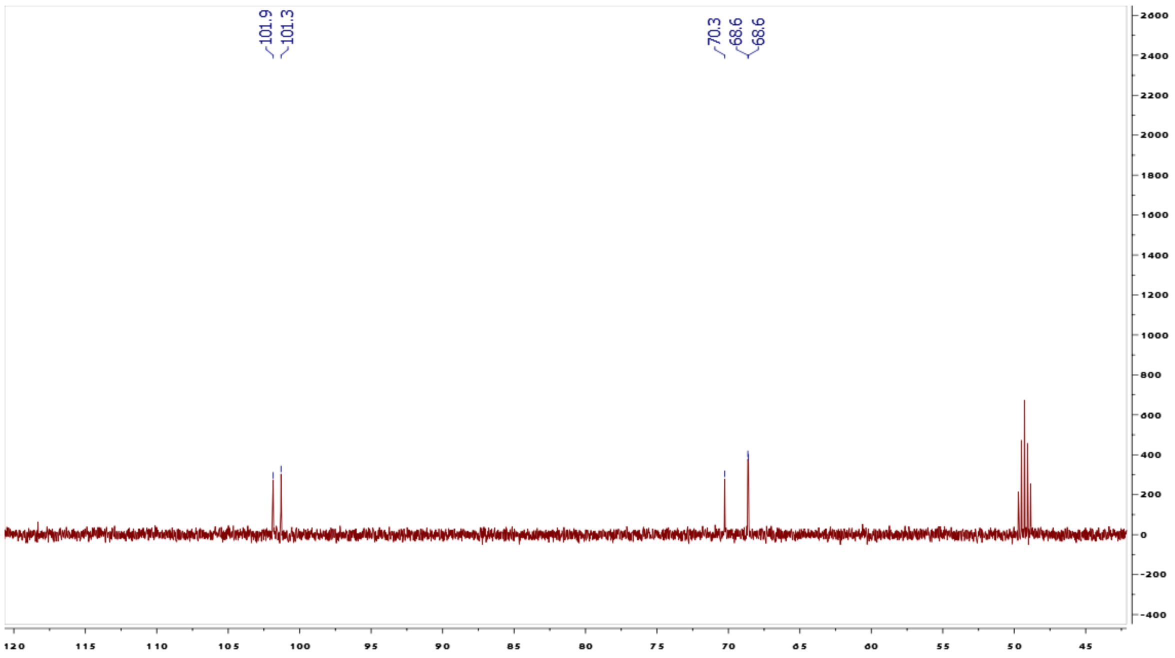

Figure S30. DEPT-135 spectrum of (4*S*, 10*R*, 4'*S*)-leptothalenone B (**5**) in CD_3OD.

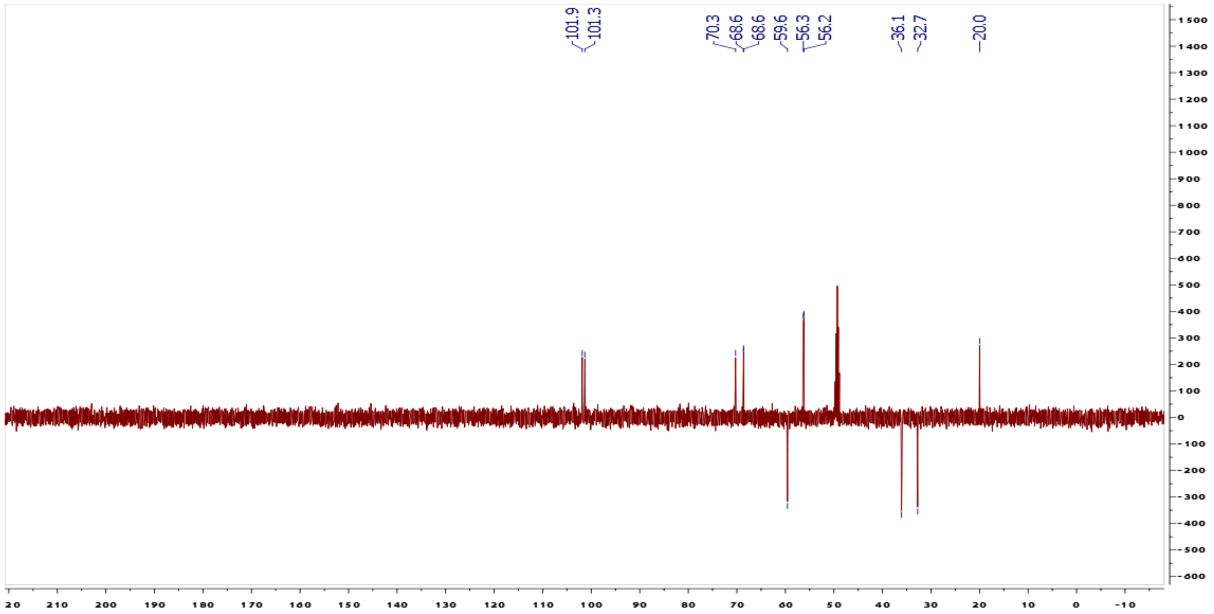

Figure S31. HSQC spectrum of (4*S*, 10*R*, 4'*S*)-leptothalenone B (**5**) in CD_3OD.

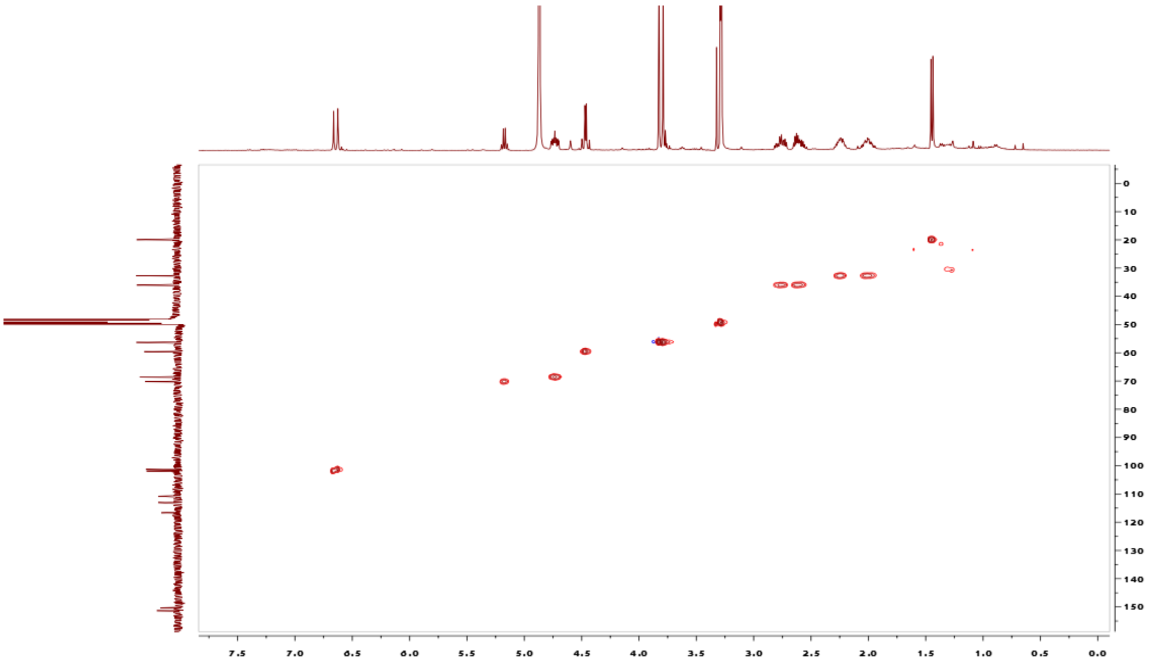

Figure S32. HMBC spectrum of (4*S*, 10*R*, 4'*S*)-leptothalenone B (**5**)in CD_3OD.

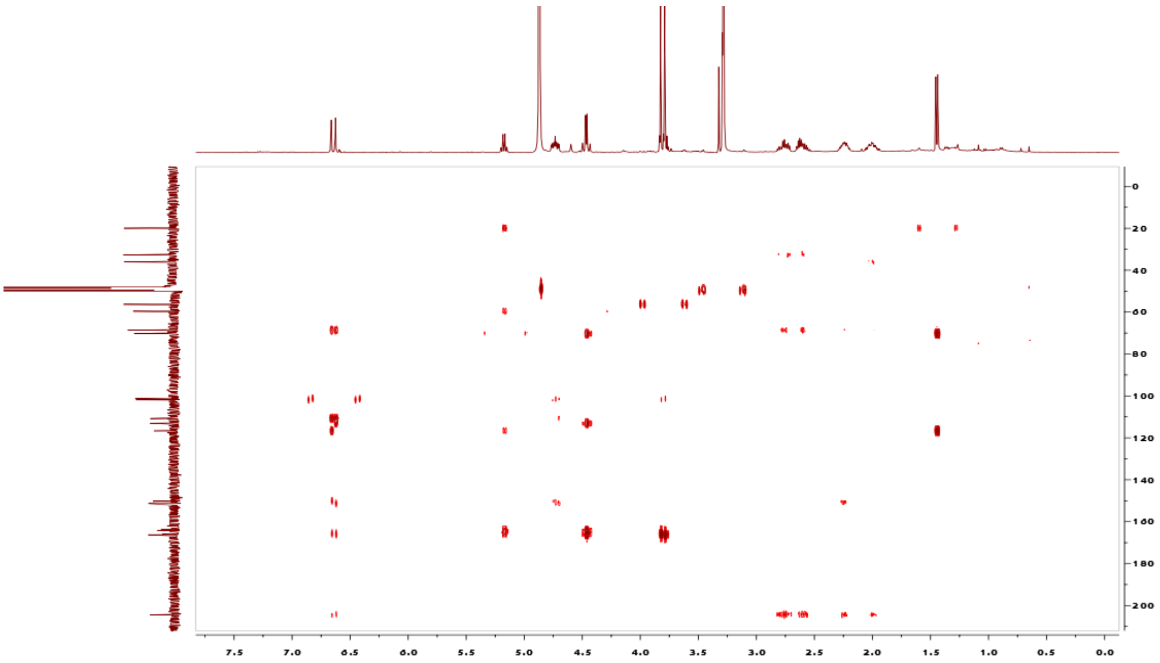

Figure S33. ^{1}H-^{1}H COSY spectrum of (4*S*, 10*R*, 4’*S*)-leptothalenone B (**5**) in CD_3OD.

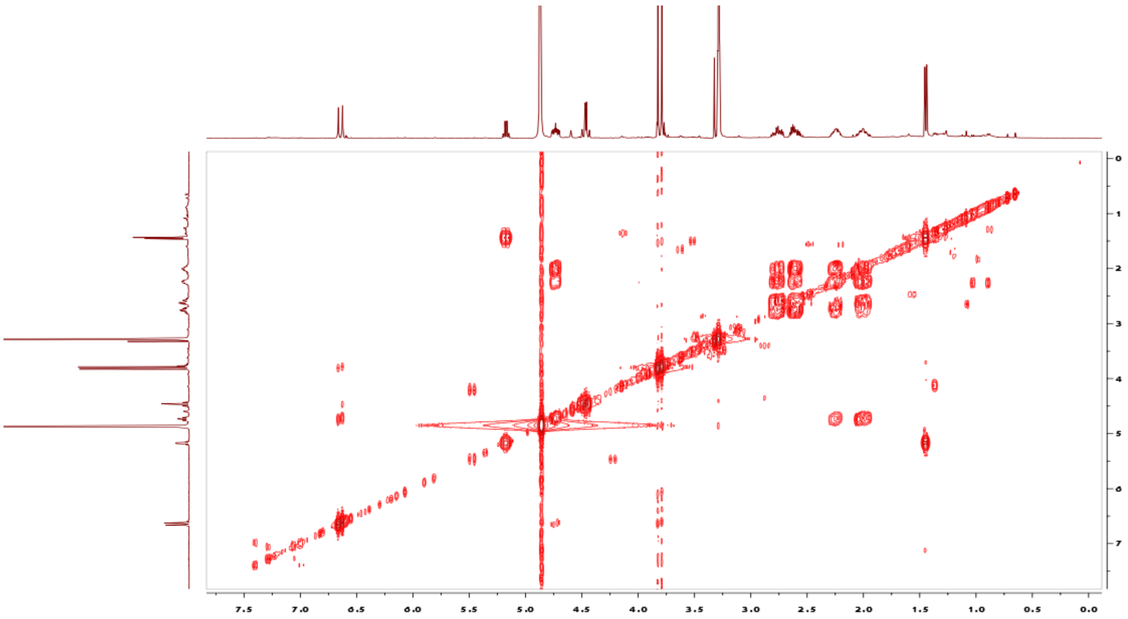

Figure S34. NOESY spectrum of (4*S*, 10*R*, 4’*S*)-leptothalenone B (**5**) in CD_3OD.

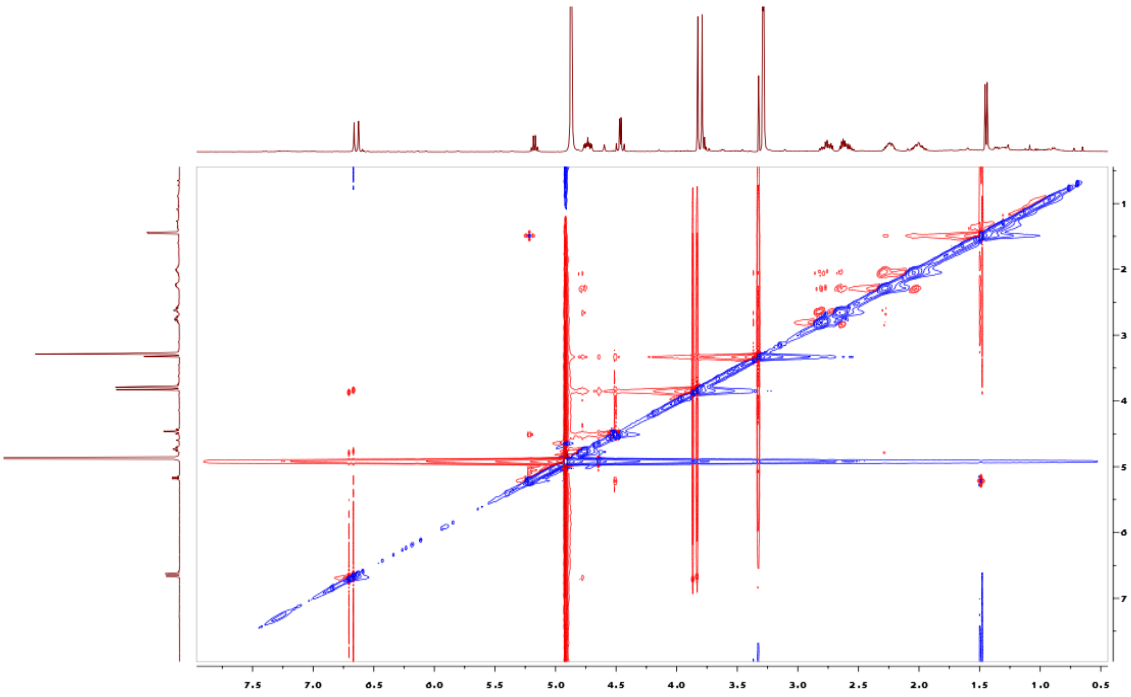

Figure S35. HR-ESIMS of (4*S*, 10*R*, 4’*S*)-leptothalenone B (**5**).

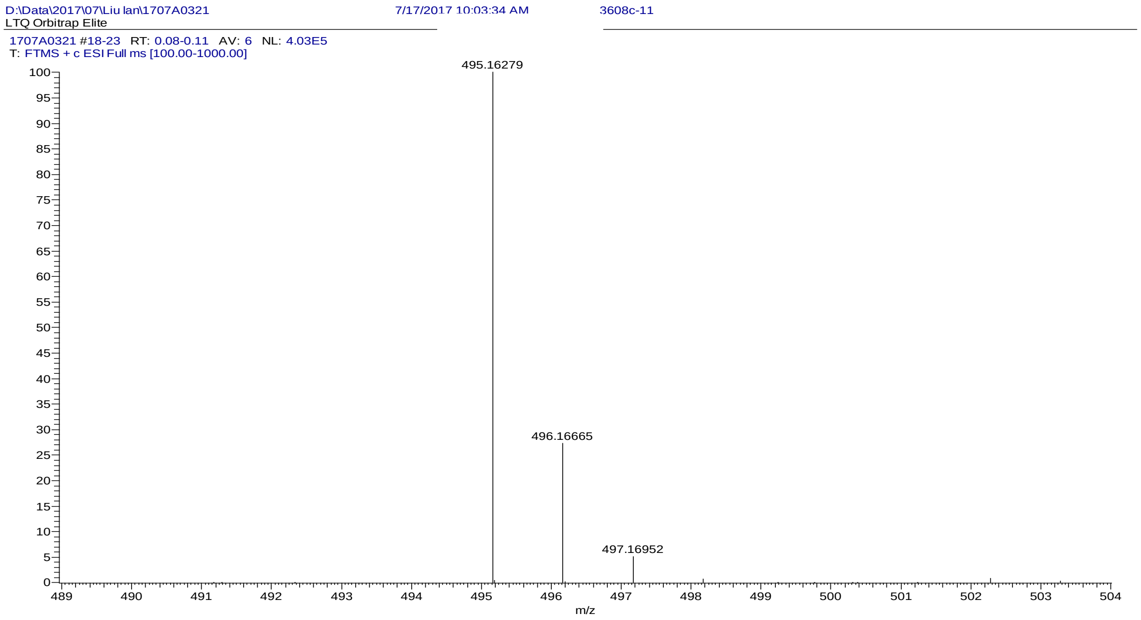

SPECTRUM - simulation :				
m/z	Theo. Mass	Delta (ppm)	RDB equiv.	Composition
495.16279	495.16255	0.48	11.5	$C_{25} H_{28} O_9 Na$

Figure S36. ^{1}H (400 MHz) NMR spectrum of (4*R*, 10*S*, 4’*S*)-leptothalenone B (**6**) in CD_3OD.

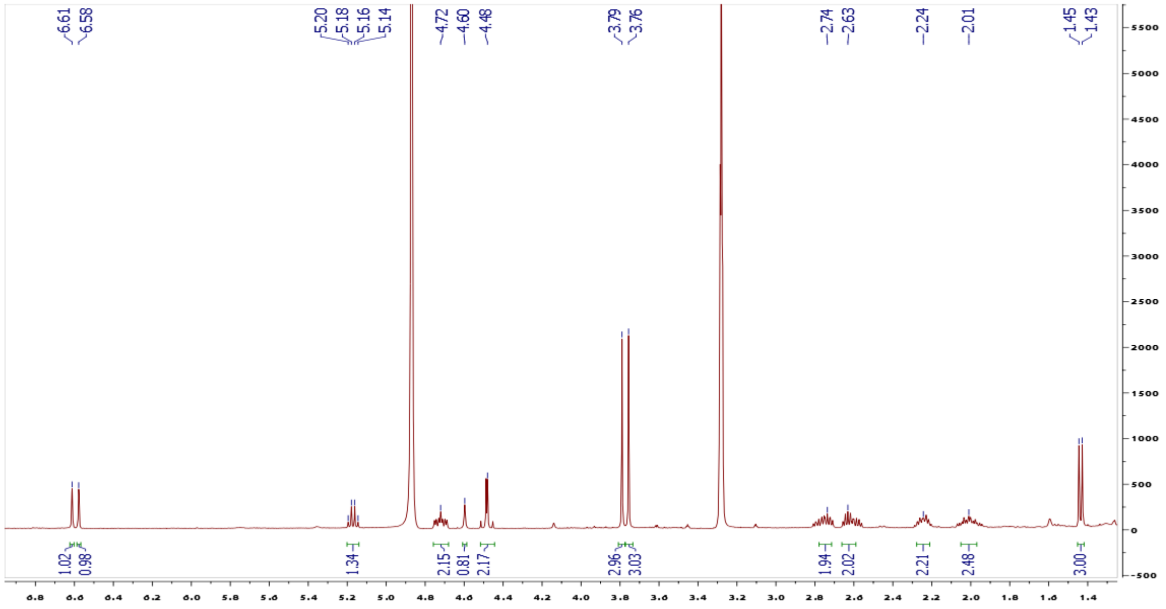

Figure S37. ^{13}C (100 MHz) NMR spectrum of (4*R*, 10*S*, 4'*S*)-leptothalenone B (**6**) in CD_3OD.

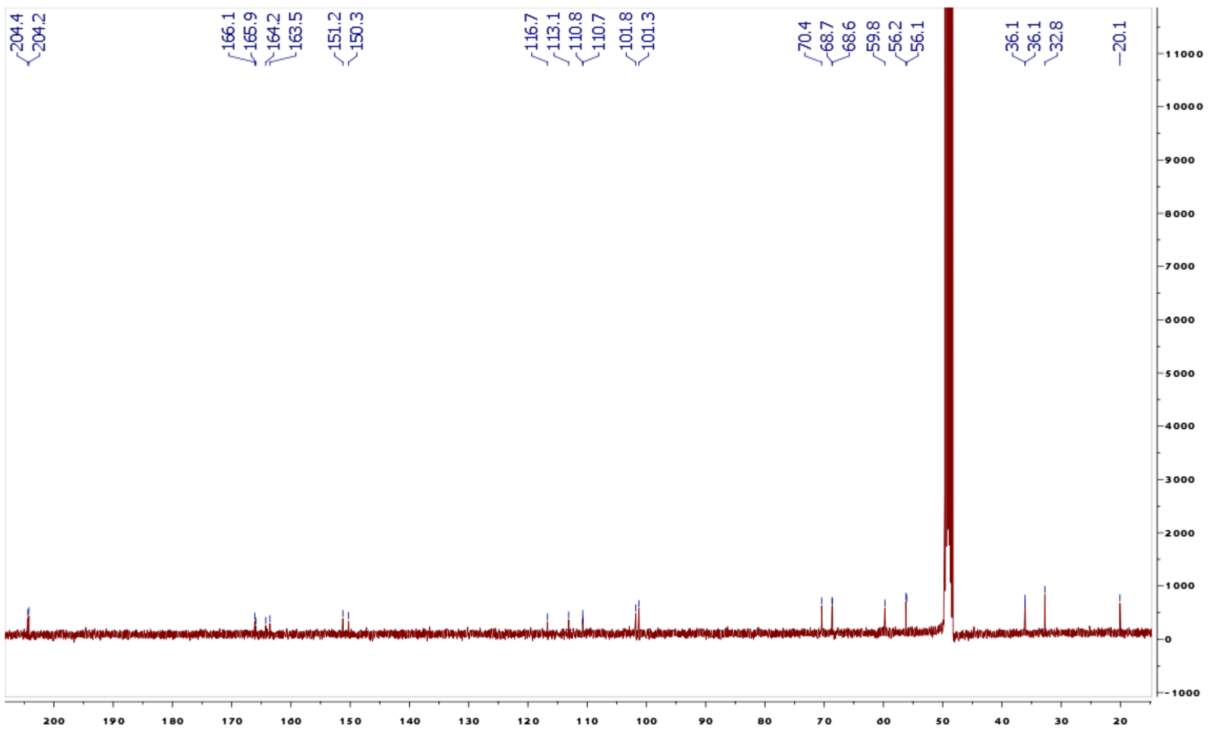

Figure S38. DEPT-90 spectrum of (4*R*, 10*S*, 4'*S*)-leptothalenone B (**6**) in CD_3OD.

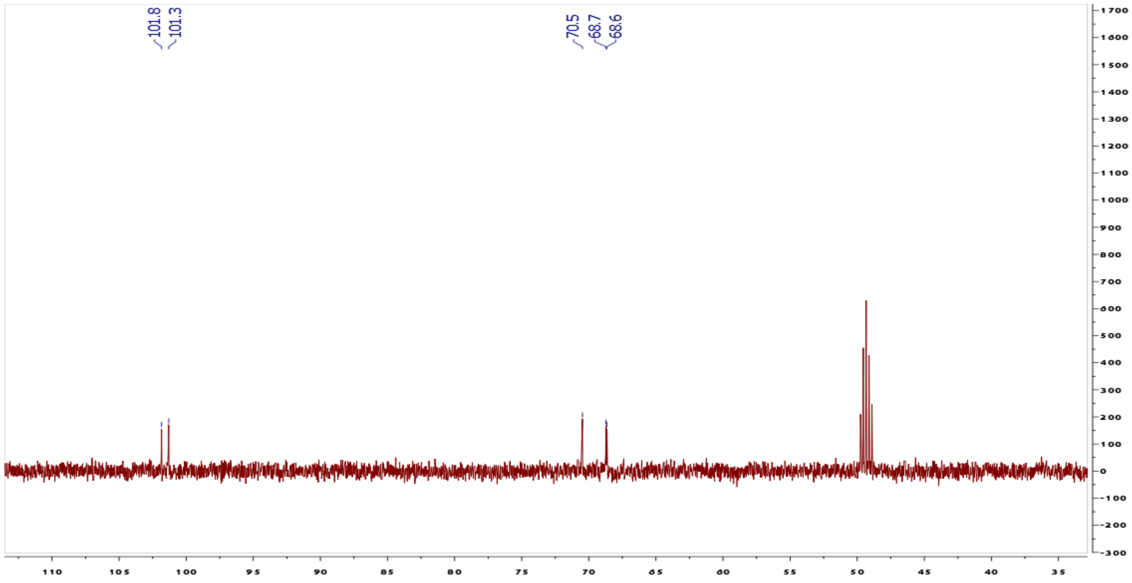

Figure S39. DEPT-135 spectrum of (4*R*, 10*S*, 4'*S*)-leptothalenone B (**6**) in CD3OD.

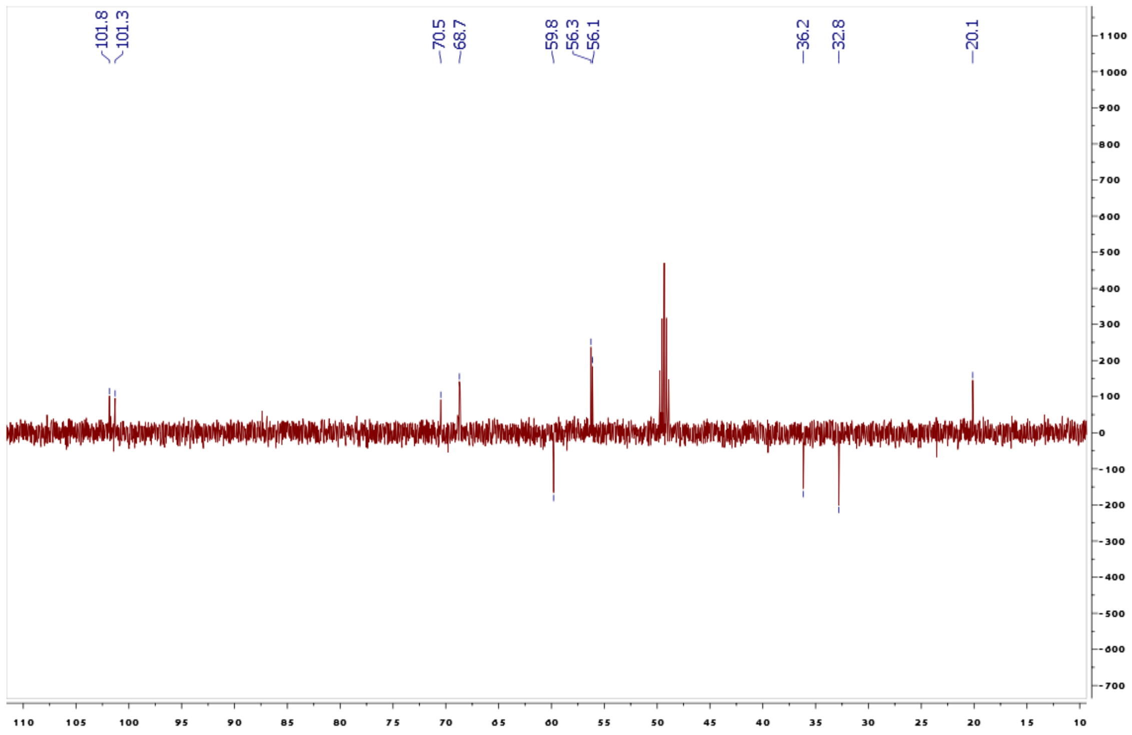

Figure S40. HSQC spectrum of (4*R*, 10*S*, 4'*S*)-leptothalenone B (**6**) in CD_3OD.

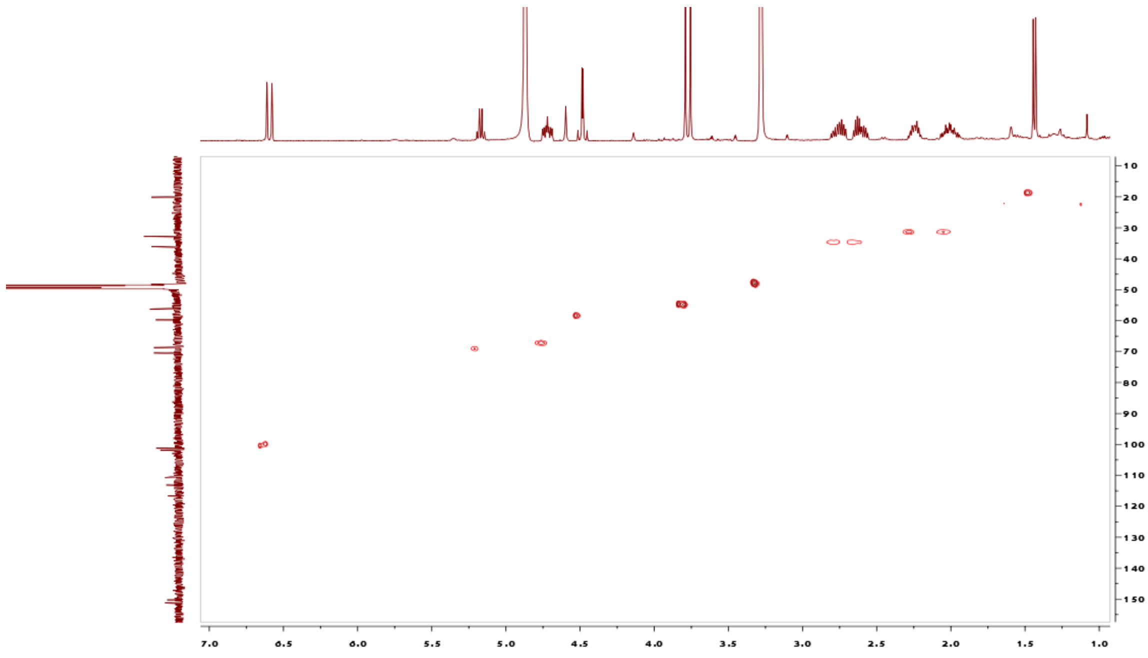

Figure S41. HMBC spectrum of (4*R*, 10*S*, 4’*S*)-leptothalenone B (**6**) in CD_3OD.

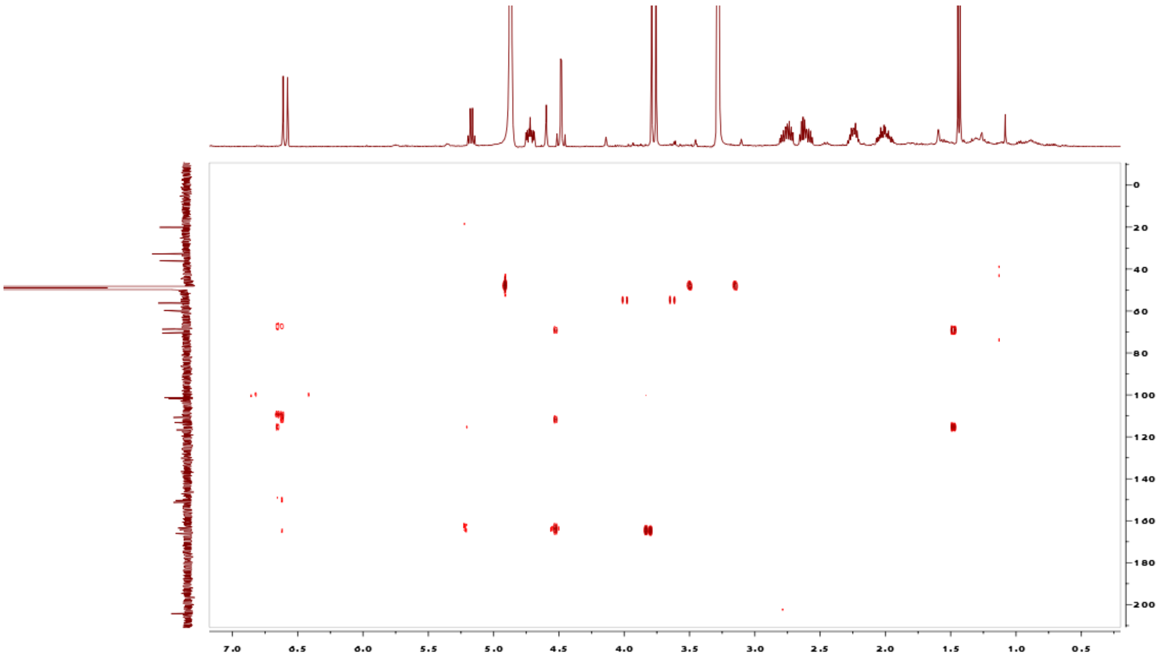

Figure S42. ^{1}H-^{1}H COSY spectrum of (4*R*, 10*S*, 4’*S*)-leptothalenone B (**6**) in CD_3OD.

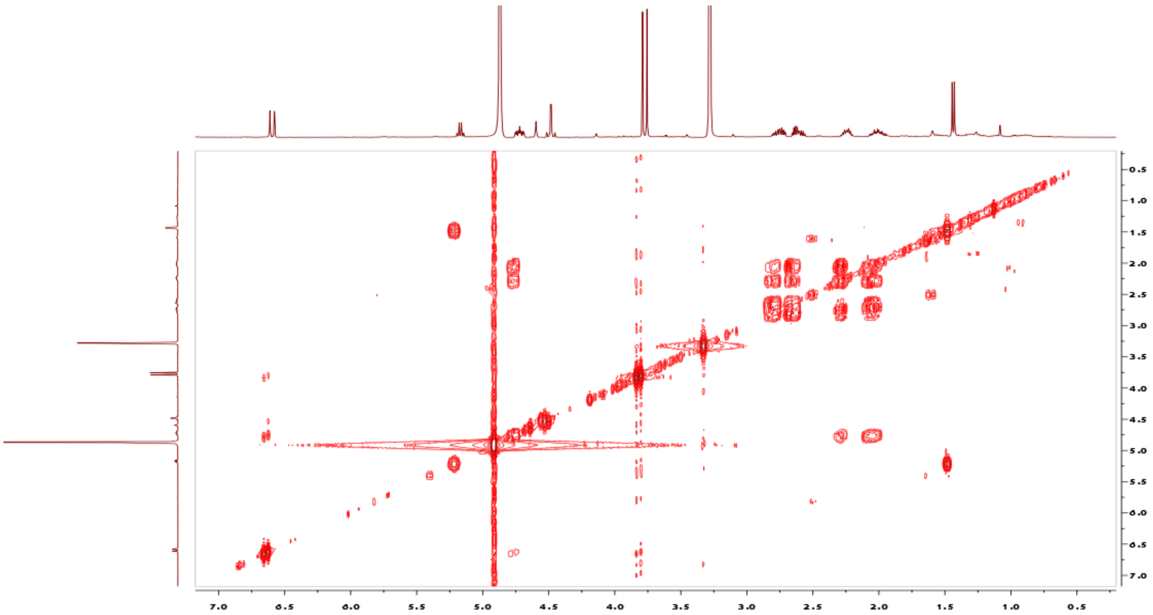

Figure S43. NOESY spectrum of (4*R*, 10*S*, 4'*S*)-leptothalenone B (**6**) in CD_3OD.

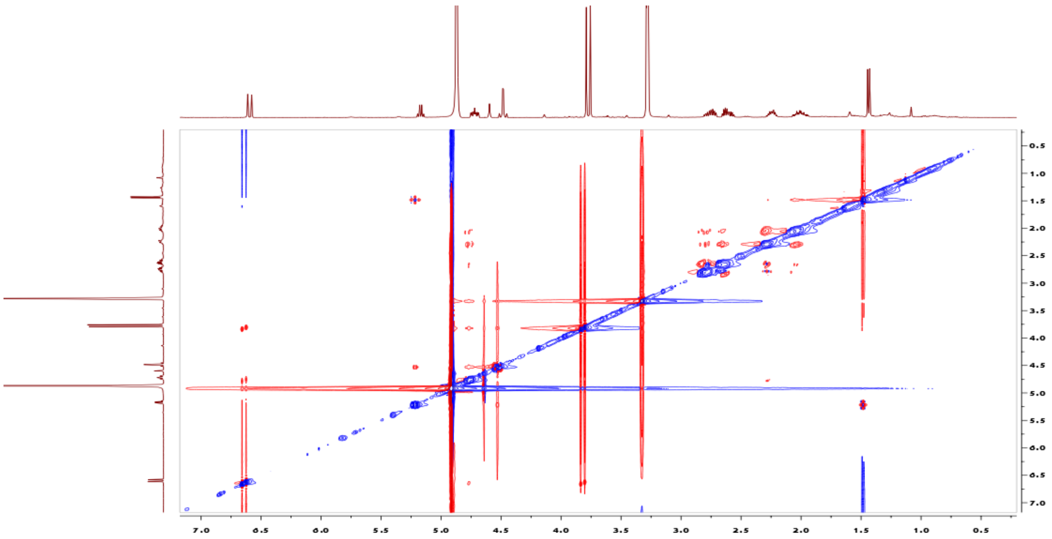

Figure S44. HR-ESIMS of (4*R*, 10*S*, 4'*S*)-leptothalenone B (**6**) in CD_3OD.

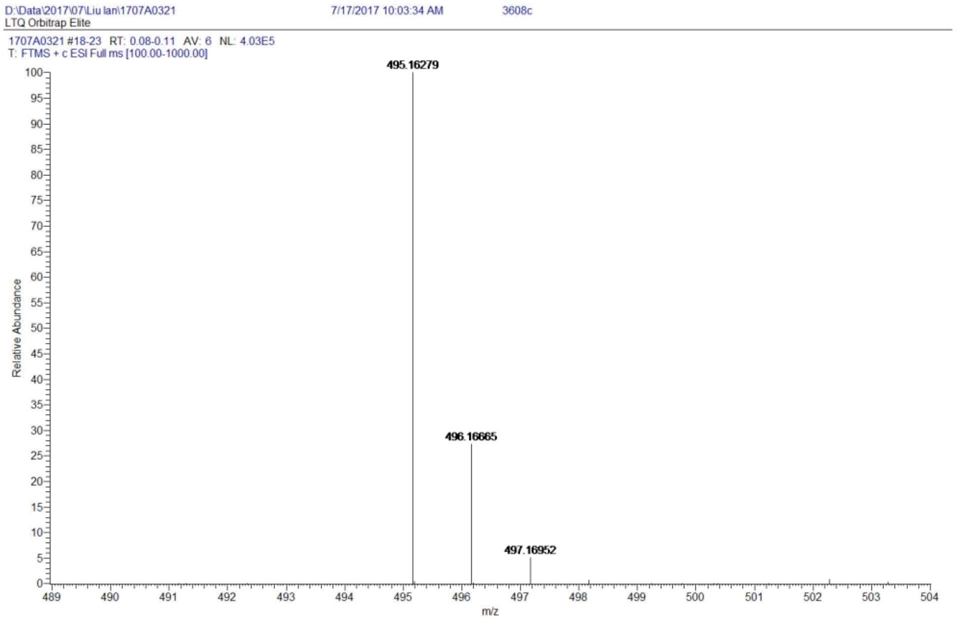

SPECTRUM - simulation :				
m/z	Theo. Mass	Delta (ppm)	RDB equiv.	Composition
495.16279	495.16255	0.48	11.5	C_{25} H_{28} O_9 Na

Figure S45. Structure of **3** resulting from single-crystal X-ray diffraction.(Single crystal X-ray crystallographic data was obtained on a Rigaku Oxford diffractometer equipped with graphite-monochromatized Cu Kα radiation at 298(2) K. Structure solution and refinement were performed with SHELXS-97, and all non-hydrogen atoms were refined anisotropically using the full-matrix least-squares method. All hydrogen atoms were positioned by geometric calculations and difference Fourier overlapping calculations. $C_{12}H_{14}O_5$, M = 238.23, orthorhombic crystal (0.40× 0.30 × 0.30 mm), bronze block, space group P212121; unit cell dimensions a = 5.298 Å, b = 12.14910 (10) Å, c = 16.69040 (10) Å, V = 1074.293 (11) Å^3; Z = 4; a total of 2163 unique reflections [R(int) = 0.0224] was measured; the final refinement gave R_1 = 0.0334, $wR2$ = 0.0885, and S = 1.121; Flack parameter = 0.00(6). Crystallographic data for the structure of **5** have been submitted to the Cambridge Crystallographic Data Centre as supplementary publication CCDC 1830777.)

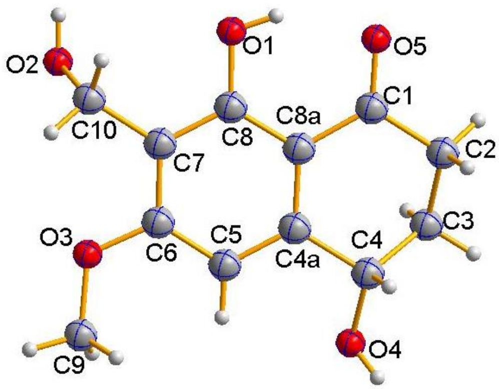

Supplement: Supplementary file 1 [file marinedrugs-16-00173-s001.pdf]
